# Bayesian Analysis of Individual Level Personality Dynamics

**DOI:** 10.3389/fpsyg.2016.01065

**Published:** 2016-07-19

**Authors:** Edward Cripps, Robert E. Wood, Nadin Beckmann, John Lau, Jens F. Beckmann, Sally Ann Cripps

**Affiliations:** ^1^School of Mathematics and Statistics, University of Western AustraliaPerth, WA, Australia; ^2^Australian Graduate School of Management, University of New South WalesSydney, NSW, Australia; ^3^School of Education, University of DurhamDurham, UK; ^4^Discipline of Business Analytics, University of SydneySydney, NSW, Australia

**Keywords:** bayesian statistics, implicit theories, mindsets, within-person, personality processes, performance spiraling, simulations

## Abstract

A Bayesian technique with analyses of within-person processes at the level of the individual is presented. The approach is used to examine whether the patterns of within-person responses on a 12-trial simulation task are consistent with the predictions of ITA theory (Dweck, 1999). ITA theory states that the performance of an individual with an entity theory of ability is more likely to spiral down following a failure experience than the performance of an individual with an incremental theory of ability. This is because entity theorists interpret failure experiences as evidence of a lack of ability which they believe is largely innate and therefore relatively fixed; whilst incremental theorists believe in the malleability of abilities and interpret failure experiences as evidence of more controllable factors such as poor strategy or lack of effort. The results of our analyses support ITA theory at both the within- and between-person levels of analyses and demonstrate the benefits of Bayesian techniques for the analysis of within-person processes. These include more formal specification of the theory and the ability to draw inferences about each individual, which allows for more nuanced interpretations of individuals within a personality category, such as differences in the individual probabilities of spiraling. While Bayesian techniques have many potential advantages for the analyses of processes at the level of the individual, ease of use is not one of them for psychologists trained in traditional frequentist statistical techniques.

## 1. Introduction

Psychological reports based on the study of between-person effects often characterize the results as relating to individual level within-person processes. For example, Blackwell et al. (2007) describe how, relative to those with an entity or fixed view, individuals with an incremental or developmental view of intelligence “display mastery-oriented strategies (effort escalation or strategy change) vs. helplessness strategies (effort withdrawal or strategy perseveration) in the face of setbacks” (Blackwell et al., 2007, p. 247). The implication for most readers is that an individual with an incremental view of intelligence will respond to an incident of failure or setback with a mastery oriented strategy, and that an individual with an entity view of intelligence will respond to an incident of failure or setback with a helplessness strategy. The argument that the views, mindsets or beliefs held by individuals shape their reactions to situations, such as failure and setbacks, has been tested for a range of latent variables, including, for example, the ideal vs. ought self (Higgins et al., 1994), learning vs. performance goal orientations (Elliott and Dweck, 1988), external vs. internal locus of control (Paulhus, 1983) and cultural group processes (Na et al., 2010). In each of these cases, the argument is made that the prior view of each individual influences his or her pattern of responses, but the effects are tested at the group level using aggregate statistics such as means, variances and correlations. Thus, statistical inferences regarding between-person differences are used to imply the existence of dynamic within-person processes.

While it is possible that the average pattern of responses observed at the group level will also be observed at the individual level, this cannot be assumed without testing at the individual level (Eysenck and Eysenck, 1985; Borsboom et al., 2003; Grice, 2015). As noted by Grice (2015, p. 1) many relationships observed at the group level do not replicate at the level of the individual, such as the structure of the Big 5 (Grice et al., 2006; Beckmann et al., 2010) and the Power Law of Learning (Heathcote et al., 2000). While this fact is widely recognized and frequently discussed (e.g., Nezlek, 2001; Schmitz, 2006), a barrier to testing models of psychological processes at the individual level has been an over reliance on the aggregate frequentist statistics of means, variances and correlations that require sample sizes greater than one (Danziger, 1990; Grice, 2015). As a result, the study of individual level processes using, for example, case studies or individual time series to capture the dynamics of within-person processes, such as those described by Blackwell et al. (2007) for entity theorists and incremental theorists, has received relatively little attention until recently.

In more recent times, the collection of individual level time series data with repeated observations of the psychological states and behaviors at multiple time points has been facilitated through the development and application of simulations (Wood et al., 2009; Beckmann et al., 2012) and experience sampling methods (e.g., Minbashian et al., 2010; Fisher and To, 2012). The analyses of these individual time series has been associated with an increased use of growth curve modeling techniques, including latent curve modeling (LCM; e.g., Goodman et al., 2011) and growth mixture models (GMM; e.g., Grimm et al., 2010), which combine LCM and finite mixture models to estimate individual trajectories. These methods provide a significant advance in the modeling of dynamic psychological processes in that, in addition to means, variances and correlations they provide estimates of the different trajectories and other features of the pattern of responses over time. However, these are frequentist methods and inference relies on the assumption of asymptotic normality of the sample estimates^1^. While this assumption is generally correct for group level estimates, it is unlikely to be true at the individual level without a large number of observations per individual. As a result, inferences at the individual level from frequentist growth curve modeling techniques are limited to point estimates and do not allow for inferences regarding dynamic within-person processes.

In the current study, we present a Bayesian approach to the modeling of individual level processes using a multiple trial task. Bayesian approaches provide greater flexibility in the modeling of the pattern of within-person processes at the individual level because they are not limited by the assumption of asymptotic normality of the distribution of sample estimates. Given a model to predict the likely observed pattern of individual level outcomes and prior assumptions regarding the parameters that describe the model, Bayesian analyses enable inferences to be made regarding each individual in a sample.

Bayesian analysis offers some advantages for psychologists interested in moving beyond group level tests of between-person differences to study if and how their theories of individual level processes impact on the observed pattern of within-person responses. First is the fact that a Bayesian approach allows for the modeling of individual processes and interpretation of the pattern of observations for each individual in a sample to see if they fit the pattern predicted by the theory. Second, the flexibility of a Bayesian approach requires a priori specification of the processes that generate observations according to the specific theory used to generate the hypotheses, including the predicted pattern of specific values for those observations. The researcher must be able to describe the dynamic model of the processes in mathematical terms, thus requiring greater precision than the prediction of a significant correlation, covariance or mean difference. Third, in the absence of significance tests, Bayesian methods require more detailed examination and explanation of the pattern of results. For example, analyses at the individual level may reveal that most but not all incremental theorists adopt a mastery strategy following failure and that most but not all entity theorists adopt a helplessness strategy. With individual level Bayesian analyses, we are able to determine how many and which individuals in each category respond in a manner that is consistent with the theoretical model and the probability that each individual responds in a manner consistent with their categorization.

In the following we will demonstrate how the Bayesian approach can be used to model within-person processes at the level of the individual. We use data from 28 professionals who worked on a complex, dynamic decision-making task and for whom we also collected data about their implicit beliefs about ability.

## 2. An example study: implicit theories of ability

Two views on intelligence were first described by Carol Dweck as implicit theories of ability (ITA) and later as mindsets (Dweck, 1999), which Dweck labeled as entity and incremental theories. Individuals with an entity theory of ability believe that intelligence is inherent or natural and therefore fixed and not readily subject to change. To the degree that experience and developmental activities make a difference, entity theorists believe it to be the result of pre-existing natural abilities. Individuals with an incremental theory of ability believe that abilities like intelligence are malleable because they are primarily the product of experience, effort and developmental activities. For an incremental theorist, natural abilities are potential to be developed and realized through developmental strategies and effort.

As noted by Blackwell et al. (2007) these two different views of intelligence have been shown to significantly influence how people react to failure and setbacks when learning new tasks (Wood and Bandura, 1989; Dweck, 1999; Tabernero and Wood, 2010). In her formulation of the ITA model, Dweck (1999) argued that entity theorists who experience failure or setbacks during learning interpret the feedback as evidence of a lack of ability and begin to doubt their capacity to learn the task. If the task is complex enough and requires full use of cognitive resources, this self-doubt interferes with subsequent performance and will lead to a downward spiral. Also, when performing at an acceptable level, entity theorists will stick with the strategy they know and not experiment with new strategies that might expose them to the risk of failure. Thus, in the early stages of learning, entity theorists will often lock into a strategy that proves suboptimal as the task unfolds. In contrast, according to Dweck (1999) those classified as incremental theorists are more likely to interpret failure feedback as evidence of a poor strategy or lack of effort. As a result of these attributions to controllable factors, incremental theorists experience less self-doubt and focus on opportunities for improvement by changing their strategy or working harder on subsequent trials, which is more likely to lead to recovery over time.

Thus, the ITA model leads to the prediction that, at an individual level, when performance drops, entity theorists are more likely to spiral further down while incremental theorists are more likely to recover. As a corollary, entity theorists are predicted to learn a task more slowly and have lower performance than incremental theorists, as has been shown at the group level (Wood and Bandura, 1989; Tabernero and Wood, 2010). As noted above, these aggregated group level results do not directly test the arguments for the differential patterns of individuals' responses to failure by entity and incremental theorists, nor do they demonstrate that the observed group level effects are the product of the predicted dynamics at the individual level. The only conclusion that can be made with confidence in comparisons of the group level learning curves of entity and incremental theorists is that entity theorists, on average, learn at a slower rate than incremental theorists. As well as allowing us to examine group or between-person differences in the average rate of performance increase (Question 1), a fuller and more direct analysis of the ITA model at the individual level using Bayesian methods also allows us to examine within-person effects (Questions 2 and 3). Our analyses address the following research questions:

1. Do individuals classified as entity theorists increase performance at a slower rate on average than individuals classified as incremental theorists?
2. Following failure what is the likelihood that an individual exhibits spiraling, that is further decreases in performance?
3. Is the probability of spiraling higher for individuals classified as entity theorists than for those classified as incremental theorists?

In addressing these questions we demonstrate features of the Bayesian approach for the analyses of individual level processes and the advantages and disadvantages of that approach. One important advantage of the Bayesian approach for the testing of psychological theories, noted above, is the requirement of specifying how the explanatory mechanisms described in the model will influence the patterns of responses for individuals, plus any assumptions built into the model. Consider research Question 2: To answer this question we need to precisely define spiraling behavior in formal mathematical terms and then develop a statistical model to test for its existence. We define spiraling behavior to be a sustained decrease in performance so that individual performance trajectories must be monotonically increasing before the commencement of any spiral and monotonically decreasing afterwards. If individuals' trajectories are assumed to be linear^2^ this means that the slopes of these trajectories are positive before and negative after the commencement of a spiral. We will show how we incorporate this structure into our model via the prior distribution of the regression coefficients.

The assumption of a prior distribution is sometimes pointed to as a subjective Achilles' heel of Bayesian methods but, in addition to the explicit statement and formal mathematical modeling of the explanatory mechanism and assumptions made, the necessity of specifying a prior distribution allows one to examine the sensitivity of any conclusions to these prior assumptions. For example, in addressing Question 3, we ask: How much prior information needs to be imposed in order to conclude that entity theorists are more likely to exhibit spiraling behavior than incremental theorists? We can make inferences about observed differences between entity and incremental theorists using prior beliefs that a difference will occur with a probability ranging from 0 to 100%. Researchers using frequentist statistics are less likely to test the sensitivity of inferences to the assumptions of their models, because the assumptions of asymptotic normality are implicit in the methods so that psychological researchers are often unaware of their existence^3^.

Another important feature of Bayesian statistics for analyzing individual level processes is that any event or quantity of interest can be treated as a random variable. In many theories of latent psychological variables that influence individual level processes of learning and performance, the situational event of interest is the experience of failure or a setback. Failures and setbacks are the result of many exogenous forces and can occur at different times for different individuals. This can be modeled as a random variable using Bayesian methods. By way of contrast, psychological experiments based on frequentist methods of inference typically seek to constrain the experience of failure to a single fixed event, a manipulation, and then use the aggregate or average group level response to infer individual responses. In Bayesian analyses, the non restrictive assumption of randomness may be applied to a parameter that describes a distribution, such as the mean slope of individual performance trajectories (Question 1), the probability that an individual will start to spiral on a given trial, or it may even be one of a set of statistical models.

These flexible features of the Bayesian approach provide two benefits for the analyses of the individual level processes in response to failure. First is that the trial on which a failure occurs does not have to be fixed but can vary randomly across trials for individuals. Thus, analyses to address Questions 2 and 3 do not have to assume that the initial experience of failure is a fixed event that occurs at the same time, or on the same trial, for all individuals in a particular group. But, when the experience of failure does occur, be it on trial 3 or trial 10, the responses of entity theorists and incremental theorists will be different. The average performance differences of entity theorists and incremental theorists, even if measured across multiple trials (e.g., Wood and Bandura, 1989), does not directly test the model proposed by Dweck (1999) and others (e.g., Blackwell et al., 2007) which describe the processes at the individual level when responding to failure events.

Relatedly, Bayesian inference based on the marginal posterior distribution accounts for the joint uncertainty surrounding all unknown parameters. This means that a statement such as “the probability that entity theorists are more likely to exhibit spiraling behavior than incremental theorists is equal to 0.95,” accounts for the uncertainty not just in the location of the commencement of the spiral, but also for the uncertainty in the size of individual and group level regression coefficients and error variances. We can therefore be more confident that the effect is real than if we were to plug-in our best guess of the other unknown parameters and compute a *p*-value.

Psychologists interested in analyzing within-person processes at the individual level will also benefit from the fact that Bayesian analyses attach probabilities to each individual's compliance and non compliance with a hypothesis, rather than just reject or accept the hypothesis at the group level. For example, research Question 2 will be answered by computing the probability of the two competing models, spiraling or no spiraling, for each individual, based on data available for all individuals. The resulting posterior probability for an individual provides an estimate of the probability that he or she will spiral on future tasks, should we wish to predict the later performance of an individual. For example, we would predict that individual A, for whom the probability of spiraling is equal to 0.99, is much more likely to spiral following failure on a future task than individual B for whom the probability of spiraling is found to equal 0.51.

By way of contrast, the frequentist approach to hypothesis testing would classify both individuals as spirallers and predict that both would spiral following failure on a future task and not differentiate between the probability of each happening. Because the observed pattern of performance for an individual will show that they either spiral or do not spiral, the probabilities of the different models included in the model averaging process must add to 1.0. For example imagine two people, individual A and individual B. For individual A the predictions for spiraling and not spiraling following failure would be weighted by 0.99 and 0.01, respectively. For individual B, the predictions for spiraling and not spiraling following failure would be weighted by 0.51 and 0.49, respectively. Clearly, there would be much greater uncertainty about the prediction for individual B than for individual A. Frequentist predictions based on model selection ignore the uncertainty associated with the model, and ignoring model uncertainty often leads to *p*-values that overstate the evidence for an effect (Hoeting et al., 1999).

As the number of possible hypotheses or models increases so do the advantages of model averaging over model selection (Raftery and Zheng, 2003). In this paper we average over a very large number of models; for each individual there are 11 possible models, the first specifying no spiral, and within the spiral hypothesis there are 10 sub models, one for each possible location of the trial on which spiraling begins, not allowing spiraling on the last two trials. Therefore, for all 28 individuals the number of possible models is 11^28^, which is very large indeed. Likelihood based model selection using frequentist procedures, such as AIC or BIC, are not feasible when the number of models under consideration is very large. With such a large number of models we use Markov Chain Monte Carlo (MCMC) methods to stochastically search across the entire model space and predictions are based on a subset of models, rather than a single model, with these predictions weighted by their posterior probability (i.e., the probability of model allocation given the data). Model averaging allows the researcher to ask questions such as “what is the probability that individual *j* started to exhibit spiraling on trial *i*?”

## 3. Methods

### 3.1. Participants

The participants were 28 managers from various organizations who were attending a 3-day executive training program at different times over a year. The 28 participants were all males and had an average age of 34.15 years (SD = 3.23 years).

### 3.2. Experimental task

The experimental task required the participants to manage a computer simulation of a small furniture production and repair workshop containing 5 workers through 12 simulated weeks of business activity (i.e., trials). In this task participants managed the performance of five employees by assigning them to each of five tasks required to complete a weekly order. The five tasks and the 5 employees remained the same throughout the 12 trials. The challenge for the participants was to learn the optimal match of employees to tasks. The employee performance norm was set at 100 at the start of the task, allowing participants to make judgments about their employees' level of performance (including increase, decrease or otherwise). Trial by trial feedback included the task performance of each of the five employees and the overall team performance. The metric for both employees and team performance was hours used as a percentage of budgeted hours for the assigned weekly order, scored so that better performance resulted in higher feedback scores. By using this feedback to test decision options systematically, managers could discover the impact of alternative choices and thereby learn how to increase the organization's performance. Therefore, for each manager there were twelve trials that recorded workgroup performance indicative of managerial ability, which we used as the dependent variable. Further details of the task are described in Wood and Bailey (1985).

The performance of workers in the simulation had two components; a deterministic component reflecting the consequence of the participant manager's decisions and a random component. The random component was included so that participants could not perfectly predict outcomes, which is a realistic representation of the business world in which managers operate. Note that we chose a dynamic computer simulation that was a novel experience for the participants, for which they had limited expertise and for which they were required to develop new strategies or adapt existing strategies (Wood and Locke, 1990). New or adapted strategies require greater cognitive effort, have a greater risk of further failure, and require greater persistence in their development and execution than well-known, routine strategies. It is these efforts that are potentially undermined by negative self-evaluations.

### 3.3. Measures

Prior to working on the furniture workshop simulation, participants completed an 8-item measure of their ITA. The eight ITA items were taken from the measures developed and validated by Dweck and her co-workers (Dweck, 1999) and included four entity type items, such as “People have a certain fixed amount of ability and they cannot do much to change it,” and four incremental type items, such as “People can always substantially change their basic skills.” All items had a 6-point Likert-type scale ranging from 1 = strongly agree to 6 = strongly disagree. The incremental items were reverse scored and the eight items were added to create a single scale (*alpha* = 0.87, *Mean* = 3.41, *SD* = 0.69), with a higher score indicating a stronger incremental theory and a lower score indicating a stronger entity theory of ability.

A median split was deemed to be an appropriate method of ITA classification as it is the method of categorization for the ITA scale used in Dweck (1999). As a result, the raw data underlying the classifications of participants based on the median split are no longer available; only the coded data has been retained. We acknowledge that using a median split is an increasingly outdated procedure. Nevertheless, we argue that our data are still informative since an individual above the median is more likely to be classified as an incremental theorist than one below the median. Furthermore, the median split provides simpler inferences, although with some loss of granularity, than a continuous variable (e.g., consider the research questions in the Introduction).

**Figure 1:**
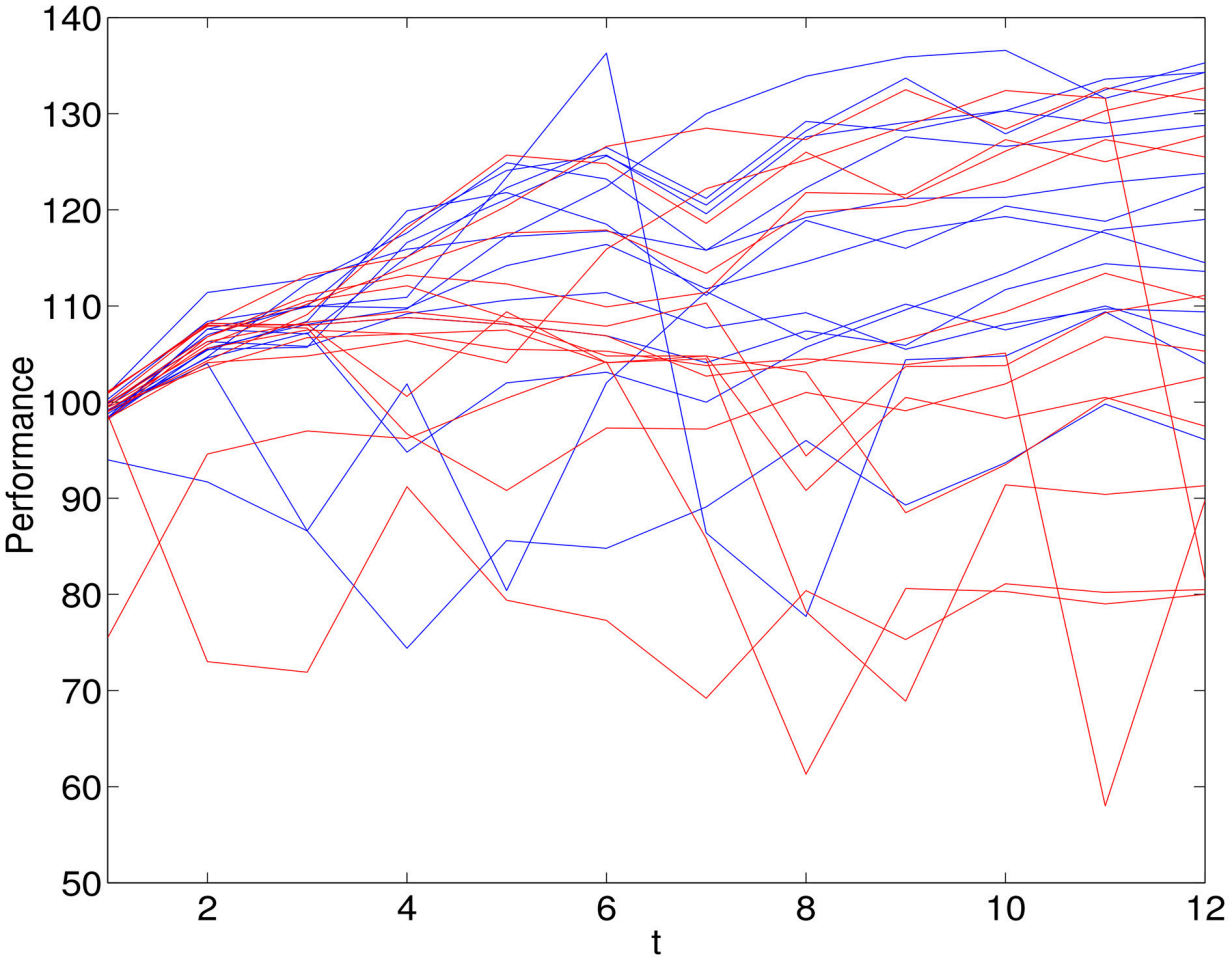
**Observations on performances over 12 trials for 14 individuals classified as entity theorists (red) and 14 individuals classified as incremental theorists (blue)**.

Based on a median split of the ITA scores, 14 individuals were classified as entity theorists and 14 classified as incremental theorists. Figure 1 shows the performance of the 28 individuals across 12 trials. Those that are classified as entity theorists are shown in red (Mean = 108.42, *SD* = 12.68) and those classified as incremental theorists are shown in blue (Mean = 112.1, *SD* = 15.04).

## 4. Bayesian hierarchical model

We start this section with a hierarchical Bayesian representation of what are commonly called latent curve models (Gelman and Pardoe, 2006; Gelman and Hill, 2007) and then demonstrate how the use of prior distributions, together with data augmentation, can be used to extend and tailor these models to answer the questions of interest to psychological researchers.

Consider a series of performance measures on *J* individuals across *T* trials. Let ***Y*** = (***y***_1._, …, ***y***_*J*._), where ***y**_j_* = (*y*_*j*1_, …, *y*_*jT*_)′ and *y*_*jt*_ is the performance of the *j*^*th*^ individual on trial *t* and denote *f*(*t*) to be some function of time. Our purpose in this paper is to demonstrate a number of features of Bayesian methods and therefore we restrict our discussion in the paper to linear functions of time with normally distributed errors. However, in Appendix A in Supplementary Material, we relax these restrictions and consider a nonlinear monotonic function of time and another error distribution.

One possible Bayesian hierarchical model is

(1.1) $$\begin{array}{l} \;y_{tj}=\alpha_{j}+\beta_{j}t+\varepsilon_{tj},\;\varepsilon_{tj}\sim N(0,\sigma^{2})\\ \alpha_{j}\sim N(\mu_{\alpha },\tau_{\alpha }^{2}),\;\beta_{j}\sim N(\mu_{\beta },\tau_{\beta }^{2}),\;\sigma^{2}\sim \text{IG}(a,b) \end{array}$$

where α_*j*_ and β_*j*_ are the regression coefficients for individual *j* and the notation IG(*a, b*) indicates an inverse gamma distribution with shape and scale parameters *a* and *b*, respectively. Model Equation (1.1) is a hierarchical one; there are trials within individuals. The model allows individuals to have different regression co-efficients and hence different expected performance trajectories, but the regression co-efficients are restricted to a distribution that depends upon parameters common to all individuals. This distribution is assumed to be normal and the parameters in common are the means, **μ** = (μ_α_, μ_β_) and variances **τ**^2^ = (τ_α_, τ_β_), of the regression coefficients. These assumptions are not necessary, but are commonly used in Bayesian methods for computational ease, and in frequentist methods because the asymptotic sampling properties of the estimators are known.

The error term in the first line of Equation (1.1) is the within-person variation and **τ**^2^ represents the between individual variation. As **τ**^2^ → (0, 0) then all individuals have exactly the same expected performance trajectory, while as **τ**^2^ → (∞, ∞) individual expected trajectories have nothing in common with each other and may as well be estimated independently. Clearly the advantage of such a model is that individual trajectories can be estimated based on only a few data points, by “borrowing" information contained in data from other individuals. Note that with only a few data points individual trajectories can only be *estimated*; *inference* surrounding individual trajectories requires the specification of a data generating process such as Equation (1.1), or a large number of data points for each individual.

The model specification is completed by specifying a prior on the hyperparameters **μ** and **τ**. In constructing these priors we use a technique known as Empirical Bayes (Robbins, 1955; Efron, 2005) where the type of prior distribution is specified by the user and then frequentist techniques are used to determine the parameters that describe these prior distributions. For example both μ_α_ and μ_β_ are assumed to be independent and normally distributed, centered around the average of the maximum likelihood estimates of the individual regression coefficients, with standard deviations equal to half the range of these quantities. See Appendix C in Supplementary Material for a full discussion.

### 4.1. Extending and tailoring the model

One of the beauties of Bayesian statistics is that, having specified the basic probabilistic data generating process, data augmentation and MCMC techniques can be used to compute the desired characteristic of any posterior distribution. In this section we show how to extend the model in the previous section to answer the research questions described in the introduction.

### 4.2. Using priors to formulate hypotheses and impose constraints

Research Question 1 is relatively straightforward to answer, so we discuss our solution to this before tackling Questions 2 and 3. In Equation (1.1) we represented a latent curve model as a hierarchical Bayes model in which the unobserved individual regression coefficients, the α's and the β's, are generated from a prior distribution. We now modify this prior to answer specific research questions. There is no reason to suppose, *a priori*, that an individual's ITA classification affects their performance before they have received any performance feedback; as argued above, it is the response to failure feedback and setbacks that differentiates entity and incremental theorists (Dweck, 1999). Therefore, we assume that the prior distribution for the intercept is the same for all individuals, $\alpha_{j}\sim N\left(\mu_{\alpha },\tau_{\alpha }^{2}\right)$. However, in order to answer research Question 1 we parameterize our prior for the slope, β_*j*_, to depend upon an individual's ITA classification. Let **μ**_β_ = (μ_*E*_, μ_*I*_)′ and let ***z***_*j*_ = (1, 0) if individual *j* is classified as an entity theorists and ***z***_*j*_ = (0, 1) otherwise. Accordingly $\beta_{j}\sim N\left(z_{j}\mu_{\beta },\tau_{\beta }^{2}\right)$, so if an individual is classified as an entity theorist then $\beta_{1}\sim N\left(\mu_{E},\tau_{\beta }^{2}\right)$, and if an individual is classified as an incremental theorist, then $\beta_{j}\sim N\left(\mu_{I},\tau_{\beta }^{2}\right)$. The difference in the mean slopes between the two classifications is given by μ_*E*_ − μ_*I*_ and Question 1 is answered by exploring the posterior distribution *p*(μ_*E*_ − μ_*I*_|**Y**); if entity theorists increase performance at a slower rate than incremental theorists then we would expect this distribution to have most of its support less than zero. Note that there is not much practical advantage in using a Bayesian method to answer research Question 1. A frequentist approach, such as restricted maximum likelihood (REML) estimation, would also suffice and we present a comparison of a frequentist and Bayesian analysis in the Results section.

Answering research Question 2 is more complex. As discussed in the introduction, the mean function must be monotonically increasing before and decreasing after the commencement of a spiral. We use the prior distributions of the regression coefficients to enforce these constraints. Suppose the regression function prior to the spiral is given by α_1*j*_ + β_1*j*_*t*, where the subscript 1 denotes the function before the spiral. If this function is monotonically increasing then the slope, β_1*j*_, must be positive. Similarly suppose the regression function after the spiral is given by α_2*j*_ + β_2*j*_*t*, then the slope, β_2*j*_, must be negative. In addition these two regression functions must intersect at the commencement of the spiral, which we call the cut point and denote by *c*_*j*_. To ensure this we need the intercept of the second regression function, α_2*j*_, to equal α_1*j*_ + *c*_*j*_(β_1*j*_ − β_2*j*_). So we have three constraints (i) β_1*j*_ > 0, (ii) β_2*j*_ < 0 and (iii) α_2*j*_ = α_*j*_ + *c*_*j*_(β_1*j*_ − β_2*j*_), all of which can be imposed in a logically consistent manner by the prior. We impose the first and second constraints by assuming that β_1*j*_ and β_2*j*_ have normal distributions constrained to be positive and negative, respectively. The third constraint is also formulated as a prior distribution, which is that the intercept α_2*j*_ is equal to α_1*j*_ + *c*_*j*_(β_1*j*_ − β_2*j*_) with probability one. Such a distribution function is referred as a Dirac delta function. Note that it is not necessary to think of the prior for α_2*j*_ as a Dirac delta function, we do so here to show that Bayesian inference is a coherent framework for imposing all model assumptions.

#### 4.2.1. Using data augmentation to model spiraling

In our response to Question 2 we not only want to identify individuals who spiral following failure but we also want to determine the likelihood of spiraling for each individual. That is, we want to be able to say, for example, that “the probability that participant 10 will exhibit spiraling behavior is 0.64.” Then, in order to address Question 3 we want to determine if the probability of spiraling behavior for each of the 28 participants is related to their categorization as an entity theorist or an incremental theorist. That is, in addition to modeling behavior at the individual level, researchers also want to understand how group level factors, such as ITA personality classification, affect these individual probabilities of spiraling. In this section we show how data augmentation can answer these questions by facilitating the MCMC scheme that performs the required multidimensional integration needed to estimate the marginal posterior distributions of interest.

To detect spiraling behavior we augment the data with a Bernoulli random variable (*Be*). For each individual we define *S*_*j*_ as

$$S_{j}=\{\begin{array}{ll} 1 & \text{if a spiral occurs at any time for individual }j,\\ 0 & \text{otherwise}. \end{array}$$

If an individual *j* exhibits spiraling behavior (i.e., *S*_*j*_ = 1) we augment the data again with another variable to indicate the point at which the spiral commences, the cut-point, *c*_*j*_, so that *c*_*j*_ = *t*|*S*_*j*_ = 1 if individual *j* begins to spiral at time *t*. The cut-point is a discrete random variable, taking values 1, …, *T* − 2 and we assume *a priori* that the spiral is equally likely to occur on any trial, therefore $\mathrm{Pr}\left(c_{j}=t|S_{j}=1\right)=\frac{1}{T-2}$. Note, under this formulation we do not allow a spiral to begin for the last two trials. The reason for this is to reduce boundary effects and to estimate the regression co-efficient with some precision.

Conditional on *S*_*j*_ and *c*_*j*_ our model for the performance score of individual *j* on trial *t* is,

if *S*_*j*_ = 1 and *t* < *c*_*j*_

$$y_{tj}\sim N(\alpha_{1j}+\beta_{1j}t,\sigma^{2}),$$

if *S*_*j*_ = 1 and *t* ≥ *c*_*j*_

$$y_{tj}\sim N(\alpha_{1j}+c_{j}(\beta_{1j}-\beta_{2j})+\beta_{2j}t,\sigma^{2})$$

with

(1.2) $$\begin{array}{l} \alpha_{1j}\sim N(\mu_{\alpha },\tau_{\alpha }^{2}),\;\beta_{1j}\sim N_{C_{+}}(z_{j}\mu_{\beta_{1}},\tau_{\beta_{1}}^{2}),\\ \;\beta_{2j}\sim N_{C_{-}}(z_{j}\mu_{\beta_{2}},\tau_{\beta_{2}}^{2}), \end{array}$$

and if *S*_*j*_ = 0 then

(1.3) $$\begin{array}{l} y_{tj}\sim N(\alpha_{1j}+\beta_{1j}t,\sigma^{2})\\ \;\alpha_{1j}\sim N(\mu_{\alpha },\tau_{\alpha }^{2}),\;\beta_{1j}\sim N_{C_{+}}(z_{j}\mu_{\beta_{1}},\tau_{\beta_{1}}^{2}),\\ \;\beta_{2j}\sim \delta (x-a) \end{array}$$

where *a* = 0.

The notations *N*_*C*_+__ and *N*_*C*_−__ indicate a normal distribution constrained to be positive and negative, respectively. The notation δ(*x*) means that δ(*x*) = 1 if *x* = 0, otherwise δ(*x*) = 0. So that, in Equation (1.3), β_2*j*_ = 0 with probability one.

**Figure 2:**
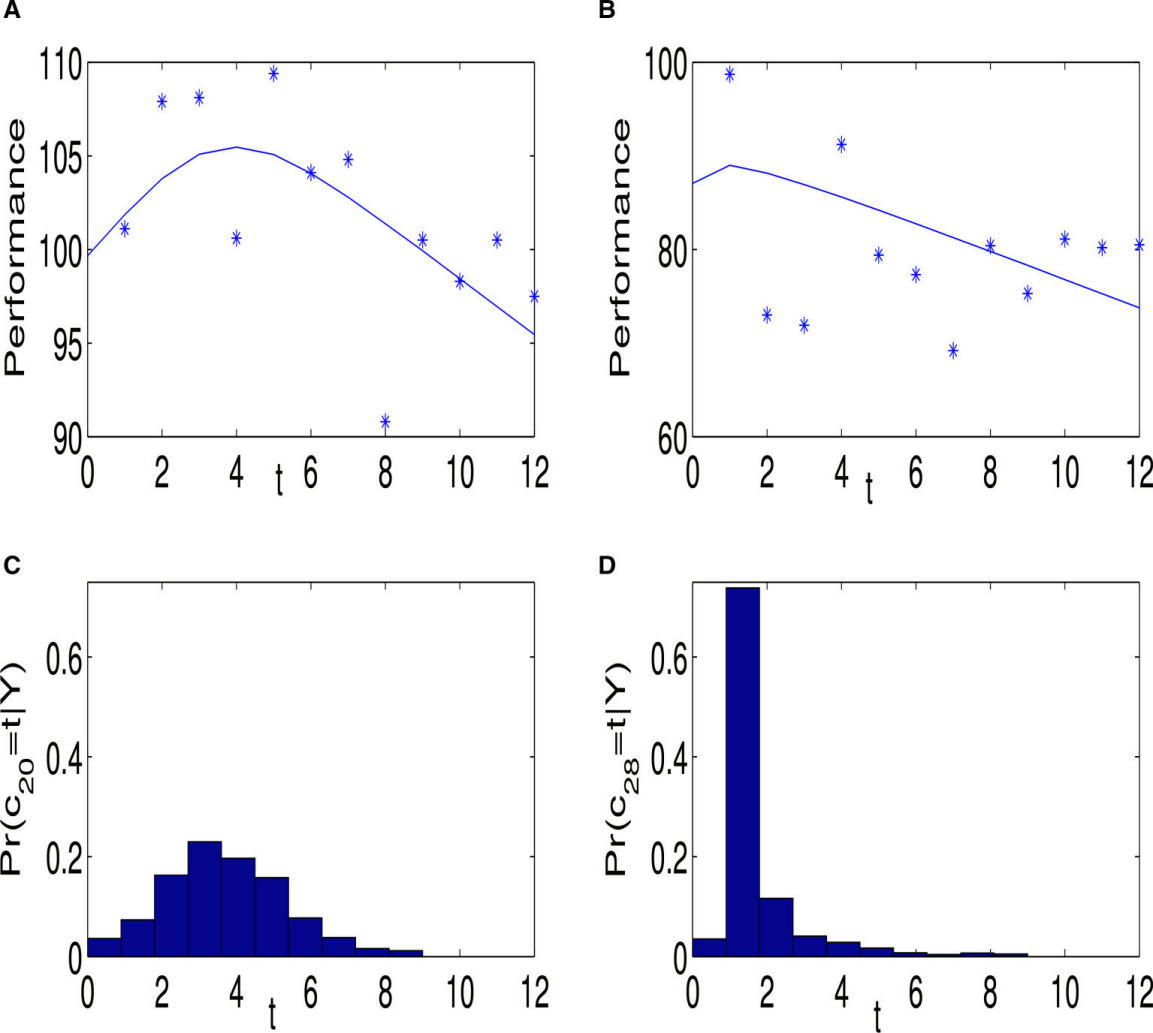
**(A)** Shows the data and fitted line for individual 20, who was classified as an entity theorist. The observed data are indicated by “^*^” and the posterior mean of the regression line is given by the blue line. **(C)** shows the posterior probability of the commencement of the spiral *c*_*j*_. **(B,D)** are corresponding plots for individual 28, who was also classified as an entity theorist.

Note that conditional on an individual spiraling and the location of the cut-point, the estimate of the expected performance trajectory is piecewise linear; α_1_ + β_1*j*_*t* before the cut point and α_1*j*_ + *c*_*j*_(β_1*j*_ − β_2*j*_) + β_2*j*_ afterwards. However, unconditional on these quantities the estimate of the mean performance trajectory is not necessarily piecewise linear. Indeed it will only be piecewise linear if the posterior probabilities of a spiral and corresponding cut-point both equal 1. Figure 2 gives an example of the performance behavior of two individuals. Figures 2A,C show the estimated posterior mean, ${\hat{E(y}}_{tj})$, and posterior probability, $\hat{\mathrm{Pr}}\left(c_{j}|Y\right)$, respectively for individual 20. Figures 2B,D are the corresponding plots for individual 28. The fit in Figure 2B is close to piece-wise linear, reflecting the fact that the posterior distribution of *c*_*j*_ is tightly centered around *t* = 1. The nonlinear fit in Figure 2A is the result of averaging across several piecewise linear functions, where the averaging is with respect to the posterior distribution of the cut-point.

We denote the probability that an individual spirals by Pr(*S*_*j*_ = 1) = π, so that *S*_*j*_ ~ *Be*(π) and research Question 2 is answered by computing Pr(*S*_*j*_ = 1|***Y***) for each individual. To answer research Question 3, we allow π to depend upon the ITA classification by modeling it as a logistic regression,

$$\pi_{j}=\frac{\exp(z_{j}\delta )}{1+\exp(z_{j}\delta )},$$

where **δ** = (δ_*E*_, δ_*I*_), so that the probability that an entity theorist spirals is $\pi_{E}=\frac{\exp\left(\delta_{E}\right)}{1+\exp\left(\delta_{E}\right)}$ and the probability that an incremental theorist spirals is $\pi_{I}=\frac{\exp\left(\delta_{I}\right)}{1+\exp\left(\delta_{I}\right)}$.

We now discuss the prior for **δ**. If we have no prior belief regarding the probabilities π_*E*_ and π_*I*_, other than they must lie between 0 and 1, then the prior on **δ** should reflect this. For example in the Appendix in Supplementary Material we use the prior **δ** ~ *N*(0, *c*_δ_***I***_2_), where ***I***_2_ is the 2 × 2 identity matrix, and show that the choice of *c*_δ_ = 4 corresponds approximately to a joint uniform prior. Having established a prior for **δ**, we answer research Question 3 by exploring the posterior distribution *p*(π_*E*_ − π_*I*_|***Y***). One way of ascertaining the strength of the relationship between the ITA personality type and the propensity to spiral is to see how strong our prior belief must be in order to conclude that there is no relationship. In the results section we show the impact of the value of *c*_δ_ has on the posterior density *p*(π_*E*_ − π_*I*_|***Y***).

Appendix D in Supplementary Material shows how data augmentation is used to facilitate the MCMC scheme that performs the multidimensional integration needed to estimate the marginal posterior distributions, *p*(μ_*E*_ − μ_*I*_|***Y***), *p*(π_*E*_ − π_*I*_|***Y***).

## 5. Results

In this section we present the results for two models; one where the possibility of spiraling is ignored and the other where it is explicitly modeled. Results are categorized as (i) results regarding parameters common to groups of individuals; (ii) results regarding specific individuals; and (iii) results regarding the effect of priors on inference. Model diagnostics, such as residual plots, and simulation results which establish the frequentist properties of the method, are contained in Appendix B in Supplementary Material.

We present here results for a linear function of time and normal distributed errors. To minimise the risk that any findings are a result of model misspecification consequent upon the choice of a particular function of time, we also obtained results for a logistic growth function, and errors that have a *t*_ν_ distribution. The results of these analyses are available in Appendix A in Supplementary Material and show that the conclusions drawn from the data are unaffected by assumptions regarding these error distributions and functions of time.

### 5.1. Results for parameters common to groups of individuals

First, we examine the results when spiraling is ignored, as described in Equation (1.1). Equation (1.1) could also be estimated under the frequentist paradigm and we did so using REML, calculated in the R package lme4 (Bates et al., 2015). Table 1 reports the results when estimating the parameters common to groups of individuals using both frequentist and Bayesian techniques. The results are very similar^4^.

**Table 1:** **Overall performance baseline (μ_α_) and performance trajectory (μ_β_) as described in Equation (1.1) and estimated by a frequentist and Bayesian analysis**.

	**Frequentist ${\hat{\mu }}_{\alpha }$**	**Bayesian ${\hat{\mu }}_{\alpha }$**	**Frequentist $\hat{\mu }\beta$**	**Bayesian ${\hat{\mu }}_{\beta }$**
Incremental theorists	101.22 (1.77)	101.2 (2.17)	1.67 (0.31)	1.67 (0.38)
Entity theorists	102.87 (1.88)	102.88 (2.21)	0.3 (0.45)	0.32 (0.55)

Standard errors and posterior standard deviations are in brackets.

**Figure 3:**
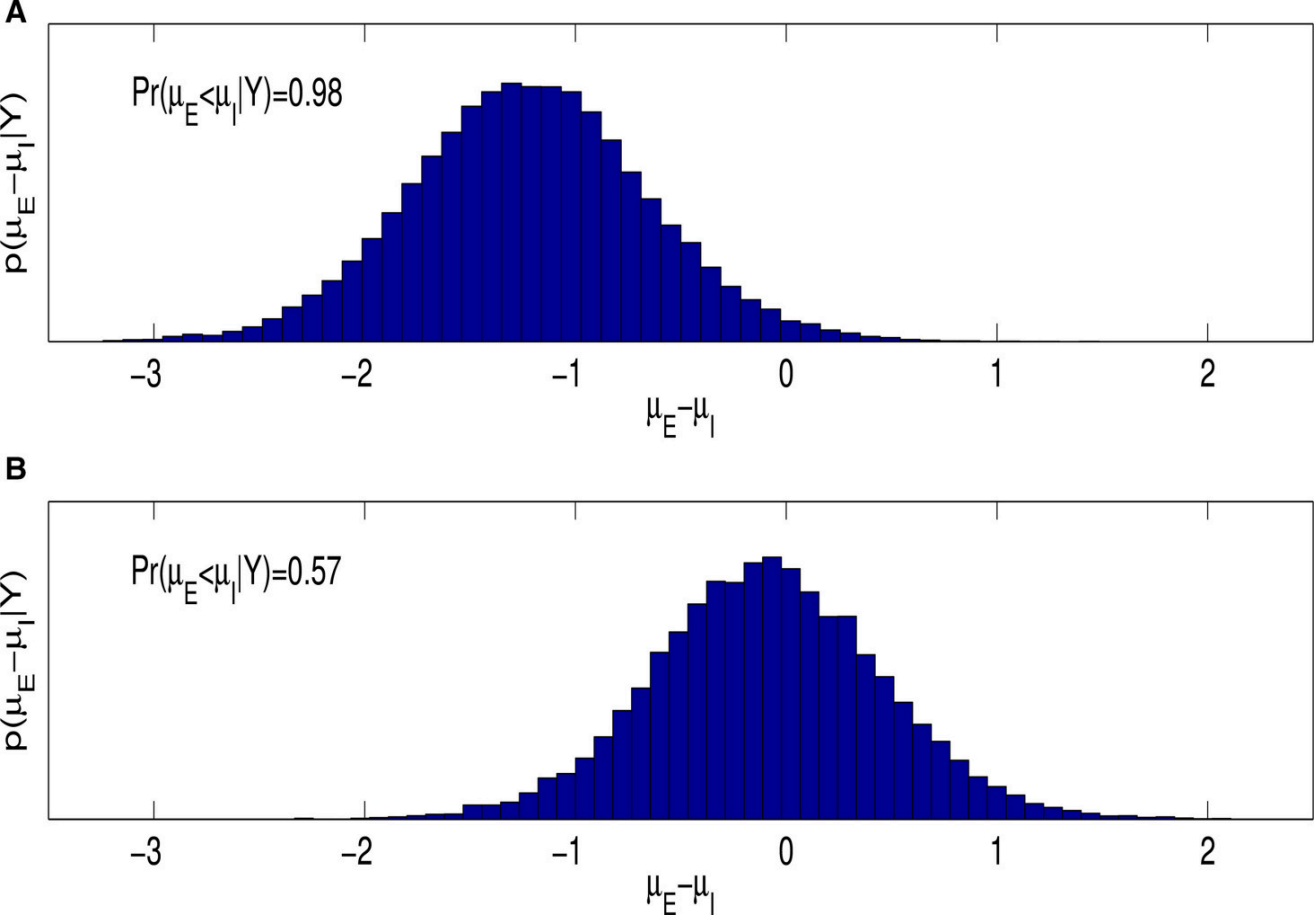
**(A)** Reports a histogram estimate of the posterior distribution of μ_*E*_ − μ_*I*_, for the model given by Equation (1.1) and *f*(*t*) = *t* and $\varepsilon_{jt}\sim N\left(0,\sigma^{2}\right)$. **(B)** is a similar plot for the model given by Equations (1.2) and (1.3).

A Bayesian analysis of Equation (1.1) also allows us to easily estimate *p*(μ_*E*_ − μ_*I*_|***Y***), the posterior distribution of the difference between the average rate of learning for entity and incremental theorists. Figure 3A is a histogram estimate of this posterior distribution and shows support for research Question 1; on average entity theorists learn more slowly than incremental theorists, with probability 0.98. In other words, given the data and prior, the probability that incremental theorists learn at a faster rate is 0.98. Figure 3A reports this by showing ~0.98 of the mass of *p*(μ_*E*_ − μ_*I*_|***Y***) lies below zero.

As noted in the Introduction, when modeling spiraling behavior explicitly in our data, as in Equations (1.2) and (1.3), a frequentist analysis is not feasible. We therefore turn our attention to Bayesian analyses only for the rest of the article. Figure 3B shows the histogram estimate of *p*(μ_*E*_ − μ_*I*_|***Y***) when the existence of spiraling is explicitly modeled. A comparison of the histograms in Figure 3 shows that the difference in the learning rate between the two ITA classifications disappears after controling for the possible existence of spiraling behavior.

**Figure 4:**
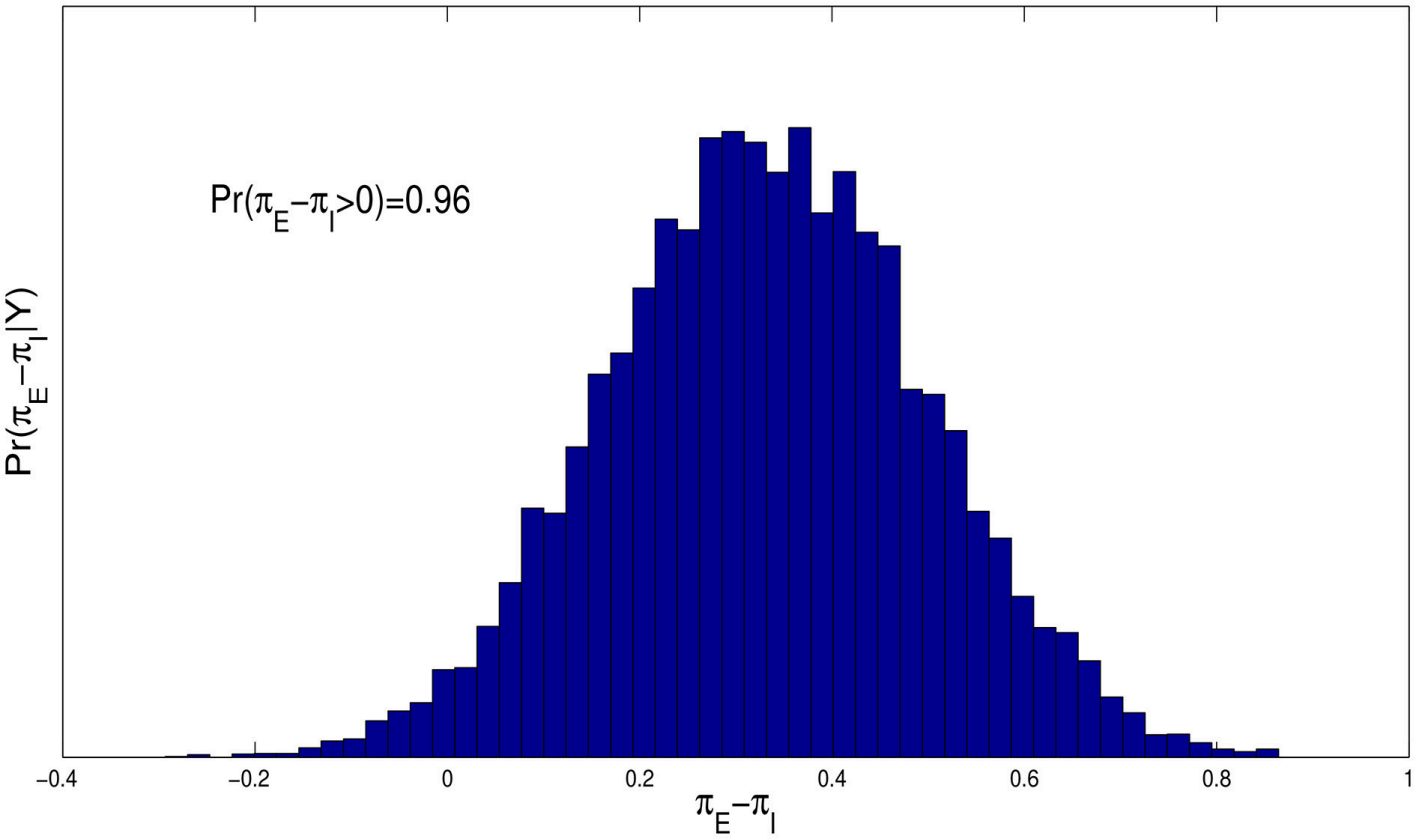
**Histogram estimate of the difference in the probability of spiraling between entity and incremental theorists, π_*E*_ − π_*I*_, for the model given by Equations (1.2) and (1.3) with *f*(*t*) = *t* and ε ~ *N*(0, σ^2^)**.

Figure 4 contains a histogram estimate of the posterior distribution, *p*(π_*E*_ − π_*I*_|***Y***), and shows that the probability of spiraling is much higher for entity theorists than for incremental theorists, with *p*(π_*E*_ > π_*I*_|***Y***) ≈ 0.96.

### 5.2. Individual level results

**Figure 5:**
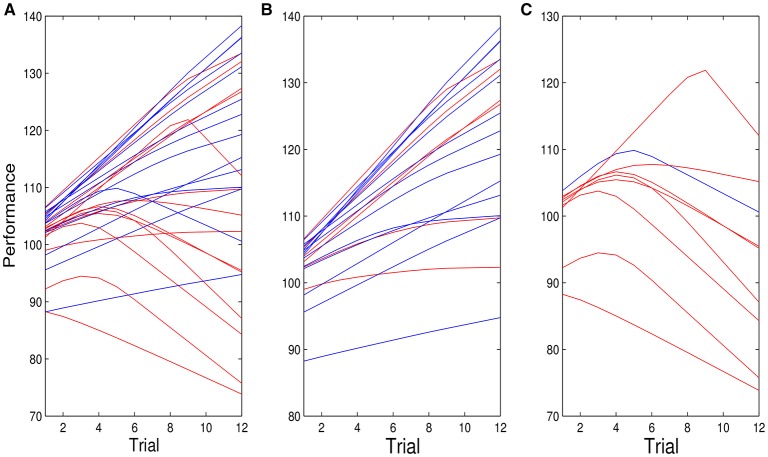
**(A)** Posterior mean of all individual performance curves for entity (red) and incremental (blue) theorists for the model given by Equations (1.2) and (1.3), *f*(*t*) = *t* and $\varepsilon_{jt}\sim N\left(0,\sigma^{2}\right)$. **(B,C)** are similar plots for individuals for whom the probability of spiraling is <0.5 **(B)** and >0.5 **(C)**.

Figure 5 shows the individual posterior mean performance trajectories for entity theorists (red) and incremental theorists (blue), for the model that allows the possibility of spiraling. Figure 5A shows the fit for all individuals. Figure 5B shows the figure for those individuals for whom the probability of spiraling was < 0.5, and Figure 5C the figure for individuals for whom the probability of spiraling was >0.5. The three panels of Figure 5 show that while entity theorists are more likely to spiral, not all do. Five out of fourteen did not. Only one out of fourteen incremental theorists exhibited spiraling behavior. Figure 5C also shows that when it is very probable that an individual spirals, the change in that individual's performance trajectory is substantial.

Table 2 shows the posterior probability of spiraling for all 28 individuals. The * and * indicate individuals classified as either an entity theorist or an incremental theorist, respectively, for whom the probability of spiraling is >0.5. An estimate of the median value of the point at which the spiral begins, ĉ_*j*_, is given in the last column. This table shows that the probability of spiraling and the point at which this spiral begins varies between individuals of the same personality classification and demonstrates the need to model behavior at the individual level.

**Table 2:** **Estimate of posterior means for individuals' probability of spiraling, $\hat{\text{p}}$r(*S*_*j*_ = 1|***Y***), and posterior medians of the commencement of the spiral, ĉ_*j*_, for all individuals classified as entity theorists (red) and as incremental theorists (blue) with *f*(*t*) = *t* and $\varepsilon_{jt}\sim N\left(0,\sigma^{2}\right)$**.

**Posterior probability of spiraling**					
Incremental theorists			Entity theorists		
Individual #	$\hat{\mathrm{Pr}}\left(S_{j}=1|Y\right)$	ĉ_*j*_	Individual #	$\hat{\mathrm{Pr}}\left(S_{j}=1|Y\right)$	ĉ_*j*_
1	0.11	0	3	0.24	0
2	0.91^*^	4	5	0.10	0
4	0.09	0	10	0.10	0
6	0.04	0	13	0.05	0
7	0.12	0	14	0.61^*^	3
8	0.18	0	16	0.33	0
9	0.04	0	18	0.97^*^	4
11	0.09	0	19	0.99^*^	9
12	0.22	0	20	0.95^*^	4
15	0.38	0	21	1.00^*^	4
17	0.14	0	22	1.00^*^	3
23	0.02	0	24	0.34	0
25	0.08	0	26	1.00^*^	3
27	0.12	0	28	0.94^*^	1
Average	0.18			0.62	

Note that for individual 19, the high probability of spiraling is a result of a low performance score on trial 12. Figure 9 in Appendix A (Supplementary Materials) demonstrates how modeling the possibility of large deviations via a t_3_ distribution mitigates the impact of outliers.

## 6. Effect of priors on results

**Figure 6:**
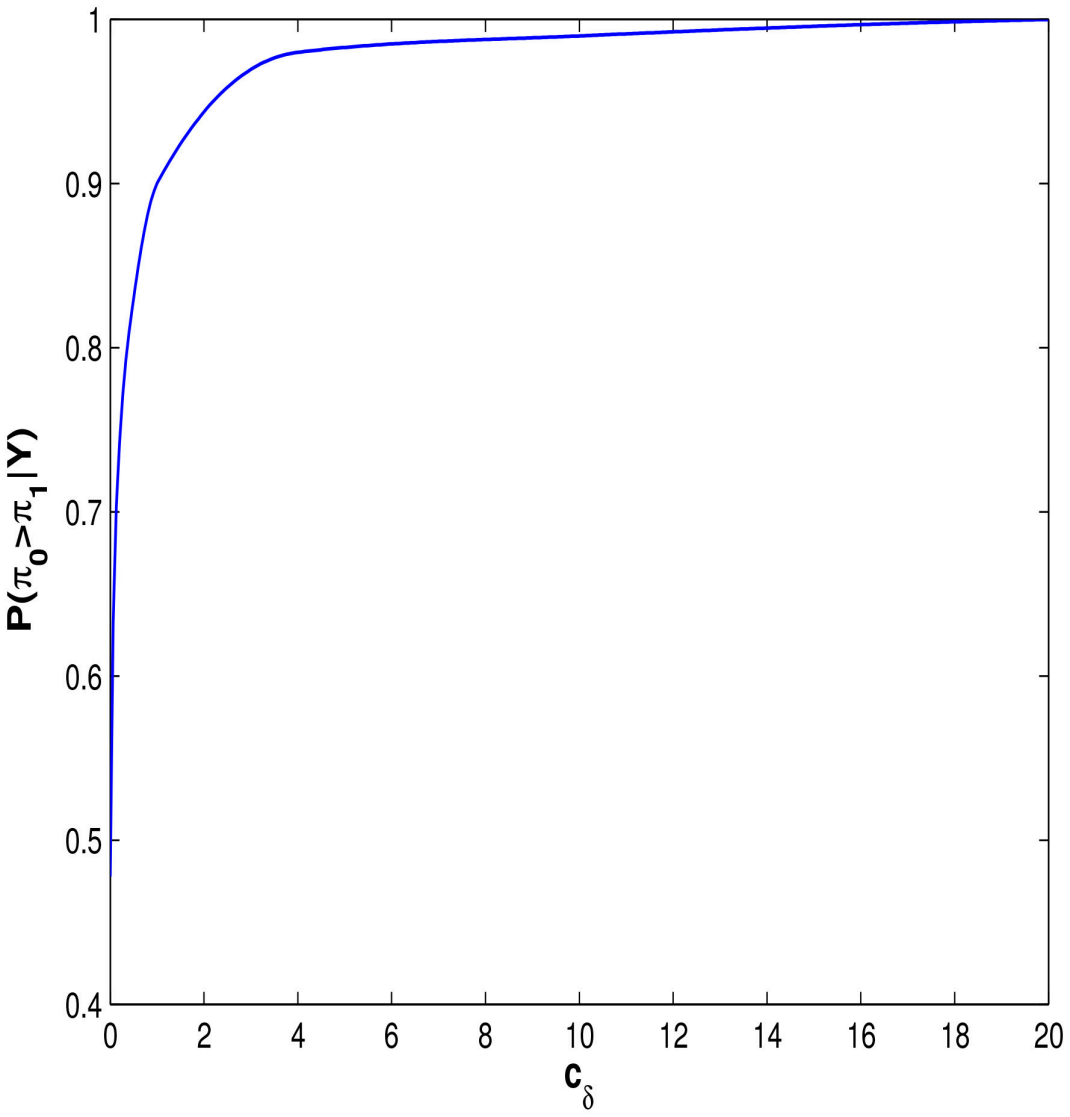
**The posterior probability that an individual classified as an entity theorist is more likely to spiral than an individual classified as an incremental theorist, as a function of the variance of the prior on **δ****.

Figure 6 shows the impact that the choice of the prior variance of **δ**, *c*_δ_, has on the posterior probability Pr(π_*E*_ > π_*I*_|***y***)). Figure 6 shows that the conclusion that entity theorists are more likely to spiral than incremental theorists is largely unchanged in the range 1 < *c*_δ_ < 20. Indeed the strength of this result can be seen by examining how much prior information needs to be imposed before the result is no longer apparent. From Figure 6 it can be seen that *c*_δ_ ≤ 0.01 before the *P*(π_*E*_ > π_*I*_|***Y***) ≤ 0.5. In other words we must be 95% certain *a priori* that the probabilities, π_*I*_ and π_*E*_, lie in the interval [0.45, 0.55], before we would conclude that, on the balance of probabilities, individuals classified as entity theorists are not more likely to spiral than those classified as incremental theorists. For a full discussion of the choice of *c*_δ_ see Appendix C in Supplementary Material.

## 7. Discussion and conclusion

In this paper we have presented a Bayesian analysis for the testing of within-person processes at the level of the individual, as well as providing the group level analyses that are usually reported in psychological research using frequentist statistical methods. The contributions and related implications of the reported study can be broken into three categories, which are discussed in turn. First, we discuss the advantages of the Bayesian method for psychologists who wish to study within-person processes at the level of the individual. Second, we discuss the results for the Bayesian analyses of the dynamic model of individual level performance outlined in the ITA model described by Dweck (1999) and the implications for testing other theories of motivation and personality at the individual level. Third, we discuss the functionality of the demands of Bayesian methods for psychologists.

The Bayesian approach provides several advantages over the more commonly used frequentist techniques for psychologists who wish to understand how within-person processes are manifest in the behavior of individuals. First, it allows inference at the individual level even when there are relatively few observations per individual, which is typically the case in longitudinal studies in personality and social psychology. In the current study, there were 12 observations per individual and we were able to test a complex dynamic model as specified by the theory. By way of contrast, if we were to rely on asymptotic arguments that underpin frequentist use of aggregate statistics for inference we would have required many more observations per person and a complex model of the type tested would require a sample of many multiples of that number. Psychological research is expensive and Bayesian methods are more efficient, as well as being more effective in enabling inferences about individuals. This is not an argument for small samples; the cost of obtaining individual level inference is that one must specify a model that generates the data and prior distributions for parameters. Like frequentist methods, Bayesian methods provide more reliable inference with larger samples. Unlike frequentist methods, Bayesian inference is based on the posterior distribution that is calculated using the observed sample. Of course, in Bayesian statistics a small sample size may mean the prior distribution has a large influence on the posterior distribution. Note, however, that one can test the effects of prior specification on the results, as was done in this study.

Second, the specification of the prior required by Bayesian methods is a formal mechanism for spelling out the assumptions and prior knowledge of the theory to be tested. This is a discipline that is not required by frequentist approaches but one that will require psychologists to think more critically about the assumptions and current state of knowledge for the theories they employ. Psychologists may not think through the assumptions that underpin the frequentist approaches that they use because there is no formal mechanism or requirement for them to do so. Over time, repeated use of Bayesian methods will begin to lead to common knowledge of priors for different theories and research questions. The current state of knowledge about a relationship can be accumulated on a study-by-study basis. Bayesian methods can also include sensitivity analyses to test for the effects of different priors on the predicted outcomes, as was shown in the results of the current study. Such sensitivity analyses can be used when there is a question about the appropriate prior or when the circumstances suggest that an established prior may not be appropriate due to, for example, challenges to an assumption. The requirement to spell out assumptions and arguments when using Bayesian methods will enable more critical assessments of the cumulative knowledge in psychological research. It will also enable more critical evaluation of populist recommendations, often espoused by consulting firms, that are based on a single study of unknown validity or relevance to the big picture.

Third, Bayesian methods enable researchers to jointly estimate the uncertainty surrounding all parameters. For example, in the current study this enabled us to treat the trial on which an individual experienced their first incident of failure that either did or did not lead to spiraling as a random variable. For psychologists seeking to predict the outcomes of individual processes, the ability to model exogenous factors, such as a performance setback, an action by another person, or some other unexpected event, as random factors, greatly enhances the validity of attempts to model the effects of those events.

This study provided the first test of the individual level performance dynamics of ITA theory. The work of Dweck and colleagues (Dweck, 1999) plus other psychologists who have used ITA theory to develop their hypotheses has been based on an argument that entity theorists respond differently to failure than incremental theorists. In particular, entity theorists are more prone to negative self-evaluations following failure than incremental theorists and these negative self-evaluations are predicted to undermine subsequent performance and lead to spiraling. The data from this study are consistent with the ITA arguments, and further studies are underway to establish the reproducibility of these findings. The results of the current study showed that those identified as entity theorists on a prior independent assessment were more likely on average to exhibit spiraling following an initial failure than those identified as incremental theorists.

We estimated the between-person effect based on the observed within-person response patterns using a bottom up, i.e., individual to group approach, rather than using group-level aggregate statistics to infer the existence of specific response patterns at the level of the individual (top down) as typically done. We also followed recent recommendations to investigate psychological phenomena as a function of time (see Roe, 2008). This enabled us to show not all individuals exhibited the outcomes predicted based on their categorization as either an entity theorist or an incremental theorist, and the onset of the spiraling behavior varied for individuals. These details, which are important for understanding the dynamics and potential limits of the theory are lost in the aggregate statistics of group level analyses. In order to capture these details, we need to model behavior at the individual level, and allow the timing of the commencement of spiraling to vary with individuals.

Approximately two-thirds of the participants classified as entity theorists exhibited spiraling behavior, while the remaining third did not. This is not an uncommon outcome for predictions based on personal characteristics, which are probabilistic and not deterministic. All assessments of the outcomes related to personality characteristics such as ITA have variability and counter indicative results that need to be explained. A further benefit of the Bayesian analyses is that it enables us to identify which of the specific participants categorized as entity theorists did not spiral. Additional knowledge of those individuals and their performance histories can then be explored to see if their deviation from the prediction of the theory are due to problems in the arguments of the theory, boundary conditions of the theory or the fact that they, for whatever reason, did not experience failure during the 12 trails of the simulations. For example, some entity theorists may not have encountered the task conditions that produce failure or they may have discovered effective strategies in the early stages of their task experience. Without the experience of failure, an entity theorist does not experience the self-doubt that can undermine their subsequent performance and may behave like an incremental theorist. Without much larger samples, current frequentist methods cannot identify the performance responses of individuals to specific events. As a result, researchers who use those methods often ignore the variability in predicted outcomes or attribute it to error. Explanations, when offered, are at the group level and refer to characteristics of the sample, the task or the context.

The fact that Bayesian techniques provide individual estimates of the probability of spiraling also has practical implications. For example, if a teacher or counselor was to provide advice to a student identified as an entity theorist, that advice would almost certainly be different for a student with a 0.95 probability of spiraling following failure in an exam than one whose probability of spiraling is found to equal to 0.51. As noted earlier, the hypothesis selection approach of frequentist statistics would label both as spirallers. The capability of social and personality psychologists to provide more nuanced, individual level analyses of individuals who vary from the mean in their assigned personality category will benefit the clinicians and practitioners who use those categories in their assessments of individuals and resulting interventions. The replication and generalization of the results in further studies will, hopefully, lead to the development of robust priors, this means *a priori* reflections regarding expected effects of tasks, performance profiles and personality constructs. Also, our results might bring spiraling as a general class of response patterns into a more process-orientated focus of attention for different psychological theories that specify differential reactions to success and failure. Another benefit of a Bayesian approach is that it allows updating of estimated probabilities as new evidence comes to hand (rather than abandon old findings and subscribing to new ones, which often is perceived by practitioners as disorientating).

Finally, we turn to the functionality of Bayesian methods for psychologists interested in the study of within-person processes at the individual level. Given the advantages outlined, we might ask why aren't more social and personality psychologists Bayesian? For established scholars whose careers have been built on the understanding and use of frequentist methods, operationalized through standardized statistical packages such as SPSS, AMOS, and Minitab, the use of Bayesian methods will present some challenges. Converting the formal mathematical model of the theory into a statistical model requires the use of a range of sampling scheme techniques, such as MCMC, Importance Sampling (IS), and Sequential Monte Carlo (SMC), to efficiently explore the entire model space. The application of these schemes is a non-trivial task and one that often requires mathematical and programming expertise (Browne and Draper, 2006). The flexibility of Bayesian methods to tailor models to answer specific problems, which is one of its strengths, makes the development of off-the-shelf standardized methods problematic. For some researchers who have not had any training in Bayesian statistics these hurdles may seem insurmountable, but not for others. Over many decades, psychology scholars have introduced increasingly sophisticated statistical methods, ranging from factor analyses to growth curve modeling. Depending upon the timing of one's career, scholars have learnt new methods either during their PhD studies or on the job. Over time the introduction of Bayesian statistics training in social sciences will, hopefully, produce a growing body of psychologists who are adept in the flexible application of Bayesian methods and there is evidence that this is a current trend (Andrews and Baguley, 2013).

Of course, not all psychologists interested in the study of dynamic individual level processes need to become experts in Bayesian techniques. Our experience in this research is that collaboration between psychologists and Bayesian statisticians can benefit both disciplines (O'Hagan et al., 2006). Scholars who develop Bayesian methods benefit because often the application of current methods to real problems leads to the development of new methods. Psychologists benefit by being able to construct formal models of their theory and to employ flexible statistical models that provide more direct individual level tests of their theory than less flexible frequentist models. In the current collaboration, the interaction with the Bayesian scholars required clear specification of the arguments and assumptions of the within-person processes in ITA theory and how they would be manifest in an observed pattern of performance over multiple trials, which were then incorporated into the formal model. The specification of the formal model led to greater clarity in the specification of the arguments for the ITA theory and the use of highly flexible Bayesian methods enabled the testing of the specified processes at the level of individuals.

Bayesian techniques have the advantage of being more adaptable for specific scientific questions than frequentist techniques. Programs such as R and Winbugs do provide pre-programmed software for some of the standard Bayesian methods used in the analyses of mixture models. However, programmed off-the-shelf software is not yet available for the Bayesian techniques used in the analyses of the complex mixture models required to address specific questions such as those addressed in this manuscript. However, the manuscript provides an explicit description of the MCMC scheme and Matlab code and data can be provided by the authors upon request. The spiraling model may well be one of a general class of models for different psychological theories that specify differential reactions to success and failure, as many social cognitive theories do. For similar, but not identical, applications we argue the collaboration between statisticians and psychologists is necessary to surmount these challenges.

## Author contributions

EC, JL, SC: contributed the development of the Bayesian methods, the data modeling and statistical analyses. RW, NB, JB: contributed the psychological conceptualization and theorizing, as well as study design and implementation.

## Funding

This research was supported by the Australian Research Council's Linkage Project Funding Scheme (project LP0669552). The views expressed herein are those of the authors and are not necessarily those of the Australian Research Council.

### Conflict of interest statement

The authors declare that the research was conducted in the absence of any commercial or financial relationships that could be construed as a potential conflict of interest.

## Footnotes

^1^The finite sample properties of the estimates in LCM and GMM have not been established.

^2^This is not a necessary assumption, but we use it as a simple example.

^3^Even when tests for finite samples exist, it is very unusual for psychological researchers to report them.

^4^We note that, for the frequentist analysis, the sample size may be inadequate for Gaussian approximations to the sampling distributions of estimators and that sampling distributions of estimators of individual level trajectories are not available.
