# Supplementary material for: Bayesian Analysis of Individual Level Personality Dynamics
Source: Front Psychol. 2016 Jul 19;7:1065. doi: 10.3389/fpsyg.2016.01065 (PMC4949490; doi:10.3389/fpsyg.2016.01065)

APPENDIX A
Alternate Models

The primary contribution of the statistical method in this article is the Bayesian two level mixture component for random effects models. Modelling this mixture structure as a function of personality type and time permits the estimation of personality group level and also individual level posterior probabilities of (a) the occurence of spiralling behavior and (b) the cut point where spiralling behavior may commence. To stay on point, the main body of the article restricts the discussion to linear mean functions, monotonic either side of the cut point, and Gaussian errors. An advantage of Bayesian methods, coupled with MCMC techniques, is the easy extension to more general models. This allows us to readily fit different models and examine the results, in order to reduce the risk that any findings are a result of model misspecification. We note immediately, in what follows, although some inference at the individual level changes, none of the essential conclusions in the main text are altered, thereby strengthening the support for the ITA.

An equivalent way of writing the two level mixture model in the model development section is for $j = 1, \dots, J$ individuals and $t = 1, \dots, T$ trials

- If $S_j = 0$
$$y_{tj} = \alpha_j + \beta_{1j} f(t) + \varepsilon_{tj}, \quad \varepsilon_{tj} \sim \text{iid} \tag{.4}$$

- If $S_j = 1$ and conditional on $c_j = t^*$
$$y_{tj} = \boldsymbol{x}_t \boldsymbol{\beta}_j + \varepsilon_{tj}, \quad \varepsilon_{tj} \sim \text{iid} \tag{.5}$$

  where $\boldsymbol{x}_t = (1, f(t) - (f(t) - f(t^*))^+, (f(t) - f(t^*))^+)$,
$$(f(t) - f(t^*))^+ = \begin{cases} f(t) - f(t^*) & \text{if} \quad f(t) - f(t^*) > 0 \\ 0 & \text{otherwise,} \end{cases}$$

  $\boldsymbol{\beta}_j = (\alpha_j, \beta_{1j}, \beta_{2j})'$ and $\varepsilon_{tj} \sim N(0, \sigma^2)$.

Now, write $z_j = (1,0)$ if individual j is an entity theorist, and $z_j = (0,1)$ if individual j is an incremental theorist. We expand the model in 4 ways to allow

1. the observational variance to be parameterized according to personality construct so that incremental and entity theorists have separate variances. That is, for each individual j,

$\sigma_j^2 = z_j(\sigma_E^2, \sigma_I^2)'$. Then if individual j is an entity theorist $\sigma_j^2 = \sigma_E^2$ and if individual j is an incremental theorist $\sigma_j^2 = \sigma_I^2$,

2. the random effects variance parameters to be parametrized according to personality construct. That is $\boldsymbol{\tau}_{\beta_1}^2 = (\tau_{\beta_{1E}}^2, \tau_{\beta_{1I}}^2)'$ and $\boldsymbol{\tau}_{\beta_2}^2 = (\tau_{\beta_{2E}}^2, \tau_{\beta_{2I}}^2)'$.

3. the error structure to have a t_3 distribution, $\varepsilon_{tj} \sim \sigma_j t_3$ to dampen the effects wide tailed error distributions or some extreme values,

4. the learning trajectory to accommodate exponential growth functions where $f(t) = 1 - \exp(-\lambda t)$ depends upon another model parameter, λ. Functions of the form $\alpha + \beta_1(1 - \exp(-\lambda t))$, are often used in the GMM literature because they have the advantage that in addition to being monotonic, an upper and lower limit exists if $\lambda > 0$. If $\beta_1 > 0$ then the lower limit is α and occurs at time $t = 0$, while the upper limit is $\alpha + \beta_1$ and occurs as $t \to \infty$. Conversely if $\beta_1 < 0$, then the upper limit is α, while $\alpha + \beta_1$ is the lower limit. The parameter λ controls the rate at which the function approaches its upper/lower limit. The rate parameters have a random effects structure so each λ_j is generated by a Gaussian distribution, the mean of which depends upon the personality classification of individual j. Also, λ_j is constrained to be positive to ensure that the upper and lower limits exist. We write this as $\lambda_j \sim N_{C_+}(z_j(\mu_{\lambda_E}, \mu_{\lambda_I})', z_j(\tau_{\lambda_E}^2, \tau_{\lambda_I}^2)')$. Then the expected performance score of individual j on trial t conditional on $S_j = 0$ becomes

$$E(y_{tj}) = \alpha_j + \beta_{1j}(1 - \exp\{-\lambda_j t\})$$

and conditional on $S_j = 1$ and $c_j = t^*$

$$E(y_{tj}) = \begin{cases} \alpha_j + \beta_{1j}(1 - \exp\{-\lambda_j t\}) & \text{if} \quad t \leq c_j \\ \alpha_j + \beta_{1j}(1 - \exp\{-\lambda_j t^*\}) + \beta_{2j}(\exp\{-\lambda_j t^*\} - \exp\{-\lambda_j t\}) & \text{if} \quad t > c_j. \end{cases}$$

For this choice of function the coefficient, β_{1j}, represents the maximum gain in performance before any possible spiral, not the rate of increase in performance. Also, since the rate which the asymptote is approached is modeled as a random effect, λ_j, the basis function is not common across individuals but rather for individual j is now $f_j(t)$.

Comparison of Results

Figure 7 contains posterior density estimates for the model with $\varepsilon_{tj} \sim \sigma_j t_3$ and $f_j(t) = 1 - \exp\{\lambda_j t\}$.

Panel (a) shows the difference in the probability of spiralling behavior between entity theorists and incremental theorists, $\pi_E - \pi_I$. Panel (a) shows that the probability of spiralling is overwhelmingly higher for entity theorists than for incremental theorists and indeed $\Pr(\pi_E - \pi_I > 0|\boldsymbol{Y}) \approx 0.98$. Panel (b) shows the difference in maximum performance gain before any possible spiral between entity and incremental theorists, $\mu_{\beta_{1E}} - \mu_{\beta_{1I}}$. Panel (b) shows that after accounting for potential spiralling behavior there exists no obvious difference in maximum gain during increasing performance between the two groups: $\Pr(\mu_{\beta_{1E}} - \mu_{\beta_{1I}} < 0|\boldsymbol{Y}) \approx 0.43$.

[FIGURE 7 about here.]

Figure 8 shows the individual fitted values when $f_j(t) = 1 - \exp\{\lambda_j t\}$ and $\varepsilon_{tj} \sim \sigma_j t_3$ and supports the results suggested by Figure 7. Figure 8 clearly shows that entity theorists are more likely to exhibit spiralling behavior. Moreover, among those individuals whose probability of spiralling is less than 0.5 (panel (a) Figure 8) there is no obvious difference in performance between entity theorists and incremental theorists. Importantly, Figures 8 and 7 support the broad conclusions of the statistical analysis in the main text regarding the ITA, suggesting model misspecification has not interfered with those aspects of the analysis.

[FIGURE 8 about here.]

Table 3 provides additional insight to differences at the individual level by reporting the posterior probability of spiralling for each individual when $f(t) = t$, $f_j(t) = 1 - \exp\{\lambda_j t\}$ and for $\varepsilon_{tj} \sim N(0, \sigma_j^2)$ and $\varepsilon_{tj} \sim \sigma_j t_3$. This table shows that the probability of spiralling varies between individuals of the same personality classification and demonstrates the need to model behavior at the individual level. Table 3 indicates those individuals who exhibit spiralling behavior – a * or * indicates an individual classified as an entity theorist or incremental theorist respectively, for whom the probability of spiralling is greater than 0.5. The results are fairly consistent, particularly for the posterior median of the cut point, although Table 3 shows different combinations of mean functions and error distributions have a stronger influence inference at the individual level than the group level.

[TABLE 3 about here.]

For instance, consider:

1. Individual 19 has a high probability of spiralling with $\hat{\Pr}(S_{19} = 1|\boldsymbol{Y}) = 0.66$ when $f_j(t) = 1 - \exp\{\lambda_j t\}$ and $\varepsilon_{tj} \sim N(0, \sigma_j^2)$. However this probability drops to 0.07 (with $\hat{c}_{19} = 0$) when $\varepsilon_{tj} \sim \sigma_j t_3$. In main article when $f(t) = t$ and $\varepsilon_{tj} \sim N(0, \sigma^2)$ then $\hat{\Pr}(S_{19} = 1|\boldsymbol{Y}) = 0.99$ and $\hat{c}_{19} = 9$. Figure 9 shows the estimated mean function for the exponential growth model with $\varepsilon_{tj} \sim N(0, \sigma_j^2)$ (dashed line) and with $\varepsilon_{tj} \sim \sigma_j t_3$ (dotted line). This figure shows that extreme observations can have a large impact on the inference regarding individual spiralling behavior. When $\varepsilon_{tj} \sim N(0, \sigma_j^2)$ the extreme observation on trial 12, shown as a '*' , resulted in the method detecting a spiral. However when the possibility of large deviations is explicitly modelled via a t_3 distribution the method does not detect spiralling behavior.

[FIGURE 9 about here.]

2. In the majority of cases the probability that an individual classified as an incremental theorist exhibits spiralling behavior decreases when the mean functions are changed from $f(t) = t$ to $f_j(t) = 1 - \exp\{\lambda_j t\}$. This is because a linear relationship between performance and trial may not be as appropriate as an exponential growth relationship. Perhaps performance increases over time at a decreasing rate and if a linear mean function is used the method occasionally interprets this decrease in the rate of improvement as the beginning of a spiral. Using an exponential growth mean function appears to correct this.

APPENDIX B
Model Diagnostics and Simulations

To check the validity of the model we report residual diagnostics and simulation results. Figure 10 shows that the residuals conform to the model assumption of a $\sigma_j \times t_3$ distribution.

[FIGURE 10 about here.]

Figure 11 displays boxplots of the posterior mean estimates of $\pi_E - \pi_I$ for 3 simulation settings of π_E and π_I, with 50 replications each. The values of π_E and π_I for each setting appear in Table 4. In all settings $\mu_{\beta_{1E}} = \mu_{\beta_{1I}} = 25$; and $\sigma_E^2 = \sigma_I^2 = 15$. These values were chosen because they are close to the posterior mean of the parameters estimated from the data.

[TABLE 4 about here.]

In the first simulation setting the probability of spiralling was zero for both entity theorists and incremental theorists. In the second setting the probability of spiralling was 0.5 for both entity theorists and incremental theorists, while in the third setting the probability of spiralling for entity theorists was set to 0.6, while for incremental theorists it was 0.1. The values of the π's for the third setting were chosen to correspond to the posterior means estimated for the real data. Data were generated from the models given by (.4) and (.5) with $\varepsilon_{tj} \sim \sigma_j \times t_3$.

Figure 11 shows that the median value of the posterior means is very close to the true value for all simulation settings. Additionally when $\pi_E = \pi_I = 0.0$ all the estimated posterior means are tightly centred around zero with an interquartile range (IQR) of [-0.02, 0.01]. However when $\pi_E = \pi_I = 0.5$, there is more variability in the posterior median estimates and the IQR is [-0.19,0.11]. This is to be expected because when spiralling behavior is not present our model detects this, and reduces to a single random effects model. However when spiralling is present, the additional uncertainty surrounding the existence and commencement of spiralling behavior induces additional variability in the parameter estimates.

In simulation setting 3, where all parameters were set to their estimated values for the real data, the boxplots show that the model estimates these parameters well, with the true parameter values very close to the median of the simulation estimates.

[FIGURE 11 about here.]

APPENDIX C
Priors

This paper uses model averaging to make inference regarding the existence of spiralling behavior. The Markov chain Monte Carlo algorithm we constructed is one of varying dimension; if spiralling behavior is not present then there is a single random effects model for performance behavior. If spiralling behavior is present, then performance behavior is described by a mixture of two random effects models, one before the spiral begins and one afterwards. Thus the dimension of the parameter space changes dependent upon which model for individual performance behavior (spiral or no spiral) is generated at each iteration. In model averaging, where the models are nested, the posterior probability of the model with the lowest dimension will be equal to one if improper priors are used, see S. A. Wood, Kohn, Shively, and Jiang (2002) and Clyde and George (2004) for a full discussion. Furthermore even if the dimension of the parameter space is fixed, placing improper priors on parameters in mixture models can result in improper posterior distributions, because there is always the possibility that no observations are allocated to a component in the mixture. For these reasons we place proper priors all parameters.

Prior for $\boldsymbol{\delta}$

Our prior for the probability of exhibiting spiralling behavior is

$$\begin{aligned} \Pr(S_j = 1) &= \frac{\exp(z_j\boldsymbol{\delta})}{1+\exp(z_j\boldsymbol{\delta})} \\ p(\boldsymbol{\delta}) &\sim N(0, c_\delta I_2), \end{aligned}$$

where the parameter c_δ determines the how much the prior shrinks the values of δ_0 and δ_1 toward zero, and hence controls the difference between an entity theorist spiralling and an incremental theorist spiralling, $\pi_E - \pi_I$. If the prior is totally uninformative, i.e. $c_\delta \to \infty$, then we are assuming that the two classifications of personality type have nothing in common regarding the existence of spiralling, and therefore may as well be analysed separately. However as the prior becomes more informative, the probability of spiralling for an individual classified as an entity theorist will approach that of an individual classified as an incremental theorist. In the extreme, if $c_\delta = 0$ then the probability of spiralling for an incremental theorist and an entity theorists will both be equal 0.5 with probability 1. Figure 12 shows the effect of c_δ has on the prior for $\boldsymbol{\pi} = (\pi_E, \pi_I)$. In panel (a), $c_\delta = 1$, in panel (b) $c_\delta = 4$ and in panel(c) $c_\delta = 10$.

[FIGURE 12 about here.]

As this figure shows placing an uninformative prior on $\boldsymbol{\delta}$, by letting $c_\delta \to \infty$ does not result in an uninformative prior for $\boldsymbol{\pi}$. As $c_\delta \to \infty$ the prior weight for π_E and π_I is concentrated on either 1 or 0. Hence choosing a large value for c_δ overstates the difference in the probability of spiralling between entity theorists and incremental theorist. Conversely choosing a small value for c_δ understates the difference in the probability of spiralling between entity theorists and incremental theorist. Choosing $c_\alpha = 4$, approximates a flat prior for π_E and π_I.

Prior for the μ's and τ^2's

We now describe the priors the random effects variances and means parametrized by their personality type. Choosing priors for variance parameters in random effects models can be tricky because of the potential for even weakly informative priors to dominate the information contained in the likelihood. For example using the proper but "non-informative" conjugate inverse gamma prior, IG(a,b), for a variance parameter, where a and b are small, will shrink the posterior distribution of the variance towards zero. For a full discussion of the effect of prior distributions for variance parameters in random effects models see (Gelman, 2006). The potential of the prior to dominate the likelihood is obviously more pronounced if the number of individuals, J, is small. This is a particular problem in this study where the number of individuals who exhibit spiralling behavior can be small. This is a particular problem in this study where the number of individuals who exhibit spiralling behavior can be as small as two or three. To mitigate the potential of the prior to dominate the likelihood we follow (Gelman, 2006) and (Browne & Draper, 2006) and place independent uniform priors on the standard deviations of the random effects $\boldsymbol{\tau} \sim U(0, a_\alpha] \times U(0, a_\beta]$. The priors on the hyperparameters are

$$
\begin{aligned}
\mu_\alpha &\sim N(g_\alpha, h_\alpha),\\
\tau_\alpha^2 &\sim \mathrm{U}(0, a_\alpha],\\
\boldsymbol{\mu}_{\beta_1} = (\mu_{\beta_{1E}}, \mu_{\beta_{1I}})' &\sim N_{C_+}\left(g_{\beta_1} \times \mathbf{1}_2, h_{\beta_1} \times \boldsymbol{I}_2\right),\\
\boldsymbol{\mu}_{\beta_2} = (\mu_{\beta_{2E}}, \mu_{\beta_{2I}})' &\sim N_{C_-}\left(g_{\beta_2} \times \mathbf{1}_2, h_{\beta_2} \times \boldsymbol{I}_2\right),\\
\boldsymbol{\tau}_{\beta_1}^2 = (\tau_{\beta_{1E}}^2, \tau_{\beta_{1I}}^2)' &\sim \mathrm{U}(0, a_{\beta_1}] \times \mathrm{U}(0, a_{\beta_1}],\\
\boldsymbol{\tau}_{\beta_2}^2 = (\tau_{\beta_{2E}}^2, \tau_{\beta_{2I}}^2)' &\sim \mathrm{U}(0, a_{\beta_2}] \times \mathrm{U}(0, a_{\beta_2}],
\end{aligned}
$$

where $\boldsymbol{I}_2$ is the 2×2 identity matrix and $\mathbf{1}_2$ is a vector of ones of length 2. If exponential growth

functions are used we have in addition

$$\begin{aligned} \lambda_j &\sim N\left((z_j(\mu_{\lambda_E},\mu_{\lambda_I})', z_j(\tau^2_{\lambda_E},\tau^2_{\lambda_I})'\right) \\ (\mu_{\lambda_E},\mu_{\lambda_I}) &\sim N(g_\lambda \times \mathbf{1}_2, h_\lambda \times \boldsymbol{I}_2) \\ (\tau^2_{\lambda_E},\tau^2_{\lambda_I}) &\sim \mathrm{U}(0,a_\lambda] \times \mathrm{U}(0,a_\lambda] \end{aligned}$$

and we adopt the following empirical Bayes approach to set the bounds:

1. If $f(t) = t$ denote the maximum likelihood estimate of the mean function coefficients for each individual (when $S_j = 0$) as $(\hat{\alpha}_j, \hat{\beta}_{1j})$ then set

$$\begin{aligned} a_\alpha &= \frac{(\max_j(\hat{\alpha}_j) - \min_j(\hat{\alpha}_j))^2}{4} \\ a_{\beta_1} &= \frac{(\max_j(\hat{\beta}_{1j}) - \min_j(\hat{\beta}_{1j}))^2}{4} \\ a_{\beta_2} &= a_{\beta_1}, \end{aligned}$$

   and $g_\alpha = \sum_{j=1}^J \hat{\alpha}_j/J$, $g_{\beta_1} = \sum_{j=1}^J \hat{\beta}_{1j}/J$, $g_{\beta_2} = -g_{\beta_1}$, $h_\alpha = a_\alpha/\sqrt{J}$, $h_{\beta_1} = a_{\beta_1}/\sqrt{J}$ and $h_{\beta_2} = a_{\beta_2}/\sqrt{J}$.

2. If $f_j(t) = 1 - \exp\{\lambda_j t\}$ denote the maximum likelihood estimate of the mean function coefficients for each individual (when $S_j = 0$) as $(\hat{\alpha}_j, \hat{\beta}_{1j}, \hat{\lambda}_j)$ then set

$$\begin{aligned} a_\alpha &= \frac{(\max_j(\hat{\alpha}_j) - \min_j(\hat{\alpha}_j))^2}{4} \\ a_{\beta_1} &= \frac{(\max_j(\hat{\beta}_{1j}) - \min_j(\hat{\beta}_{1j}))^2}{4} \\ a_{\beta_2} &= a_{\beta_1}, \\ a_\lambda &= \frac{(\max_j(\hat{\lambda}_j) - \min_j(\hat{\lambda}_j))^2}{4} \end{aligned}$$

   and $g_\alpha = \sum_{j=1}^J \hat{\alpha}_j/J$, $g_{\beta_1} = \sum_{j=1}^J \hat{\beta}_{1j}/J$, $g_{\beta_2} = -g_{\beta_1}$, $g_\lambda = \sum_{j=1}^J \hat{\lambda}_j/J$, $h_\alpha = a_\alpha/\sqrt{J}$, $h_{\beta_1} = a_{\beta_1}/\sqrt{J}$ and $h_{\beta_2} = a_{\beta_2}/\sqrt{J}$ and $h_\lambda = a_\lambda/\sqrt{J}$.

Prior for σ^2

We set an uninformative uniform prior for the observational variances contained in $\boldsymbol{\sigma}^2$. That is, $p(\sigma_E) \sim U(0,k]$ and $\sigma_I \sim U(0,k]$ for some large non-negative constant k.

APPENDIX D
Sampling Scheme

Write $\boldsymbol{S} = (S_1, S_2, \ldots, S_J)$, $\boldsymbol{C} = (c_1, c_2, \ldots, c_J)$ and for the case when $S_j = 0$

$$\boldsymbol{X}_{j|0} = \begin{bmatrix} 1 & f(1) \\ 1 & f(2) \\ \vdots & \vdots \\ 1 & f(T) \end{bmatrix} \quad \text{and} \quad \boldsymbol{b}_{j|0} = (\alpha_j, \beta_{1j})'$$

and for the case when $S_j = 1$ and conditioned on $c_j = t^*$

$$\boldsymbol{X}_{j|1} = \begin{bmatrix} 1 & f(1) - (f(1) - f(t^*))^+ & (f(1) - f(t^*))^+ \\ 1 & f(2) - (f(2) - f(t^*))^+ & (f(2) - f(c_t^*))^+ \\ \vdots & \vdots & \vdots \\ 1 & f(T) - (f(T) - f(t^*))^+ & (f(T) - f(t^*))^+ \end{bmatrix} \quad \text{and} \quad \boldsymbol{b}_{j|1} = (\alpha_j, \beta_{1j}, \beta_{2j})'.$$

Also, write $\boldsymbol{b}_0 = \{\boldsymbol{b}_{j|0} : S_j = 0\}$, $\boldsymbol{b}_1 = \{b_{j|1} : S_j = 1\}$ and $\boldsymbol{B} = \{\boldsymbol{\alpha}, \boldsymbol{b}_1\}$, $\boldsymbol{\Theta} = (\Theta_0, \Theta_1)$, $\Theta_0 = \{\mu_\alpha, \tau^2_\alpha, \mu_{\beta_{1E}}, \mu_{\beta_{1I}}, \tau^2_{\beta_{1E}}, \tau^2_{\beta_{1I}}\} = \{\mu_\alpha, \tau^2_\alpha, \boldsymbol{\mu}_{\beta_1}, \boldsymbol{\tau}^2_{\beta_1}\}$,
$\Theta_1 = \{\mu_\alpha, \tau^2_\alpha, \mu_{\beta_{1E}}, \mu_{\beta_{1I}}, \tau^2_{\beta_{1E}}, \tau^2_{\beta_{1I}}, \mu_{\beta_{2E}}, \mu_{\beta_{2I}}, \tau^2_{\beta_{2E}}, \tau^2_{\beta_{2I}}\} = \{\mu_\alpha, \tau^2_\alpha, \boldsymbol{\mu}_{\beta_1}, \boldsymbol{\tau}^2_{\beta_1}, \boldsymbol{\mu}_{\beta_2}, \boldsymbol{\tau}^2_{\beta_2}\}$. Finally, to implement the MCMC scheme when the ε_{tj}'s have a scaled t_3 distribution, define $\varepsilon_{tj} = e_{tj}\sqrt{3/(\kappa_{tj})}$, where $\kappa_{tj} \sim \chi^2_3$ and $e_{tj} \sim N(0, \sigma^2)$. Then conditional on κ_{tj} the distribution of $\varepsilon_{tj}|\kappa_{tj}$ is $N(0, \omega_{tj})$ where $\omega_{tj} = \sigma^2 3/\kappa_{tj}$, and ω_{tj} is the tth, diagonal element of a diagonal matrix Ω_j. Finally, write $\boldsymbol{\Omega} = \{\Omega_j : j = 1, 2, \ldots, J\}$.

The sampling scheme is then

1. Sample $\boldsymbol{S}$.

$$p(\boldsymbol{S}|\boldsymbol{Y}, \boldsymbol{\Theta}, \boldsymbol{\Omega}) = \prod_{j=1}^{J} p(S_j|\boldsymbol{y}_j, \Omega_j, \boldsymbol{\Theta})$$

where

$$p(S_j = 1|\boldsymbol{y}_j, \Omega_j, \Theta_1) = \frac{p(\boldsymbol{y}_j|\Omega_j, \Theta_1, S_j = 1)P(S_j = 1)}{p(\boldsymbol{y}_j|\Omega_j, \Theta_1, S_j = 1)P(S_j = 1) + p(\boldsymbol{y}_j|\Omega_j, \Theta_0, S_j = 0)P(S_j = 0)}$$

and

$$\begin{aligned} p(\boldsymbol{y}_j|\Omega_j,\Theta_1,S_j=1) &= \frac{\sum_{t=1}^{T-2}}{T-2}\times \\ &\int_{\mathbb{R}\times C_+\times C_-} p(\boldsymbol{y}_j|S_j{=}1,\Omega_j,\Theta_1,c_j{=}t,\boldsymbol{b}_{j|1})p(\boldsymbol{b}_{j|1}|\Theta_1,S_j{=}1)d\boldsymbol{b}_{j|1}\Pr(c_j{=}t|S_j{=}1) \\ p(\boldsymbol{y}_j|\Omega_j,\Theta_0,S_j=0) &= \int_{\mathbb{R}\times C_+} p(\boldsymbol{y}_j|S_j{=}0,\Omega_j,\Theta_0,\boldsymbol{b}_{j|0})p(\boldsymbol{b}_{j|0}|\Theta_0)d\boldsymbol{b}_{j|0} \end{aligned} \tag{.6}$$

The integrals in (.6) are equal to

(a)

$$\begin{aligned} p(\boldsymbol{y}_j|\Omega_j,\Theta_0,S_j=0) &= \frac{|\boldsymbol{T}^*_{j|0}|^{1/2}}{(2\pi)^{T/2}|\boldsymbol{T}_{j|0}|^{1/2}|\Omega_j|^{1/2}} \\ &\quad\times\exp\left\{-\frac{1}{2}\left(\boldsymbol{y}_j'\Omega_j^{-1}\boldsymbol{y}_j+\boldsymbol{M}'_{j|0}\boldsymbol{T}^{-1}_{j|0}\boldsymbol{M}_{j|0}-\boldsymbol{M}^{*\prime}_{j|0}\boldsymbol{T}^{*-1}_{j|0}\boldsymbol{M}^*_{j|0}\right)\right\} \\ &\quad\times\frac{1-\Phi\left((\infty,0)'|\boldsymbol{M}^*_{j|0},\boldsymbol{T}^*_{j|0}\right)}{1-\Phi\left((\infty,0)'|\boldsymbol{M}_{j|0},\boldsymbol{T}_{j|0}\right)} \end{aligned}$$

where

$$\boldsymbol{T}_{j|0}=\begin{bmatrix}\tau^2_\alpha & 0\\ 0 & z_j\boldsymbol{\tau}^2_{\beta_1}\end{bmatrix}, \qquad \boldsymbol{M}_{j|0}=\begin{bmatrix}\mu_\alpha\\ z_j\boldsymbol{\mu}_{\beta_1}\end{bmatrix},$$

$$\boldsymbol{T}^*_{j|0}=\left(\boldsymbol{X}'_{j|0}\Omega_j^{-1}\boldsymbol{X}_{j|0}+\boldsymbol{T}^{-1}_{j|0}\right)^{-1} \quad\text{and}\quad \boldsymbol{M}^*_{j|0}=\boldsymbol{T}^*_{j|0}\left(\boldsymbol{X}'_{1|0}\Omega_j^{-1}\boldsymbol{y}_j+\boldsymbol{T}^{-1}_{j|0}\boldsymbol{M}_{j|0}\right)$$

(b) and

$$\begin{aligned} p(\boldsymbol{y}_j|\Omega_j,\Theta_1,c_j=t,S_j=1) &= \frac{|\boldsymbol{T}^*_{j|1}|^{1/2}|}{(2\pi)^{T/2}|\boldsymbol{T}_{j|1}|^{1/2}|\Omega_j|^{1/2}} \\ &\quad\times\exp\left\{-\frac{1}{2}\left(\boldsymbol{y}_j'\Omega_j^{-1}\boldsymbol{y}_j+\boldsymbol{M}'_{j|1}\boldsymbol{T}^{-1}_{j|1}\boldsymbol{M}_{j|1}-\boldsymbol{M}^{*\prime}_{j|1}\boldsymbol{T}^{*-1}_{j|1}\boldsymbol{M}^*_{j|1}\right)\right\} \\ &\quad\times\frac{\Phi\left((\infty,\infty,0)'|\boldsymbol{M}^*_{1|j},\boldsymbol{T}^*_{1|j}\right)-\Phi\left((\infty,0,0)'|\boldsymbol{M}^*_{1|j},\boldsymbol{T}^*_{1|j}\right)}{\Phi\left((\infty,\infty,0)'|\boldsymbol{M}_{1|j},\boldsymbol{T}_{1|j}\right)-\Phi\left((\infty,0,0)'|\boldsymbol{M}_{1|j},\boldsymbol{T}_{1|j}\right)} \end{aligned}$$

where

$$\boldsymbol{T}_{j|1} = \begin{bmatrix} \tau_\alpha^2 & 0 & 0 \\ 0 & z_j\tau_{\beta_1}^2 & 0 \\ 0 & 0 & z_j\tau_{\beta_2}^2 \end{bmatrix}, \qquad \boldsymbol{M}_{j|1} = \begin{bmatrix} \mu_\alpha \\ z_j\mu_{\beta_1} \\ z_j\mu_{\beta_2} \end{bmatrix},$$

$$\boldsymbol{T}^*_{j|1} = \left(\boldsymbol{X}'_{j|1}\Omega_j^{-1}\boldsymbol{X}_{j|1} + \boldsymbol{T}^{-1}_{j|1}\right)^{-1} \quad \text{and} \quad \boldsymbol{M}^*_{j|1} = \boldsymbol{T}^*_{j|1}\left(\boldsymbol{X}'_{j|1}\Omega_j^{-1}\boldsymbol{y}_j + \boldsymbol{T}^{-1}_{j|1}\boldsymbol{M}_{j|1}\right)$$

2. Sample $\boldsymbol{C}$.

Draw $\boldsymbol{C}$ from

$$p(\boldsymbol{C}|\boldsymbol{Y},\boldsymbol{\Theta},\boldsymbol{S},\boldsymbol{\Omega}) = \prod_{j=1}^{J} p(c_j = t|\boldsymbol{\Theta},\boldsymbol{y}_j,S_j,\Omega_j)$$

If $S_j = 0$, c_j no sampling is required. Conditional on $S_j = 1$, c_j is drawn according to

$$p(c_j = t|\boldsymbol{\Theta},\boldsymbol{y}_j,S_j = 1) = \frac{\frac{1}{T-2}p(\boldsymbol{y}_j|\Theta_1,c_j = t,S_j = 1,\Omega_j)}{\sum_{t'=1}^{T-2}\frac{1}{T-2}p(\boldsymbol{y}_j|\Theta_1,c_j = t',S_j = 1,\Omega_j)}$$

where the densities in the denominator and numerator are given in step 1.

3. Sample $\boldsymbol{B}$.

Draw $\boldsymbol{B}$ from

$$p(\boldsymbol{B}|\boldsymbol{Y},\boldsymbol{\Theta},\boldsymbol{S},\boldsymbol{C},\boldsymbol{\Omega}) = \prod_{j:S_j=0} p(\boldsymbol{b}_{j|0}|\boldsymbol{y}_j,\Theta_0,S_j = 0,\Omega_j)\prod_{j:S_j=1} p(\boldsymbol{b}_{j|1}|\boldsymbol{y}_j,\Theta_1,S_j = 1,c_j = t,\Omega_j)$$

Again, from step 1 we can see that $\boldsymbol{b}_{j|0}$ is drawn according to $N(\boldsymbol{M}^*_{j|0},\boldsymbol{T}^*_{j|0})$ restricted to the region $\mathbb{R}\times C_+$ and $\boldsymbol{b}_{j|1}$ is sampled according to $N(\boldsymbol{M}^*_{j|1},\boldsymbol{T}^*_{j|1})$ restricted to the region $\mathbb{R}\times C_+\times C_-$. To draw $\boldsymbol{b}_{j|0}$ and $\boldsymbol{b}_{j|1}$ we note that linear transformations of truncated normal vectors, and the one-dimensional conditional distributions, are also truncated normal (Rodriguez-Yam, Davis, & Scharf, 2004), so that drawing the elements of $\boldsymbol{b}_{j|0}$ and $\boldsymbol{b}_{j|1}$, reduces to drawing a sequence of one-dimensional constrained conditional normal distributions.

4. Sample $\boldsymbol{\lambda}$.

If the basis functions are exponential growth curves then draw $\boldsymbol{\lambda} = (\lambda_1, \ldots, \lambda_J)$, from

$$\begin{aligned} p(\boldsymbol{\lambda}|\boldsymbol{Y},\boldsymbol{S},\boldsymbol{C},\boldsymbol{B},\boldsymbol{\Omega},\mu_\lambda,\tau^2_\lambda) &= \prod_{j=1}^{J} p(\lambda_j|\boldsymbol{y}_j, S_j = s_j, c_j, \boldsymbol{b}_{j|s_j}, \Omega_j, \mu_\lambda, \tau^2_\lambda) \\ &= \prod_{j=1}^{J} p(\boldsymbol{y}_j|\lambda_j, S_j = s_j, c_j, \boldsymbol{b}_{j|s_j}, \Omega_j) p(\lambda_j|\mu_\lambda, \tau^2_\lambda) \end{aligned}$$

using a Metropolis-Hastings step. If the current value of λ_j in the chain is λ^c_j then a new value, λ^N_j, is drawn from a proposal density $q(\lambda_j) \sim N_{C_\lambda}(\hat{\lambda}_j, \hat{\Sigma}_{\lambda_j})$. The value of $\hat{\lambda}_j$ is the value that maximizes $l(\lambda_j)$ where $l(\lambda_j) = \log(p(\boldsymbol{y}_j|\lambda_j, S_j = s_j, c_j, \boldsymbol{b}_{j|s_j}, \Omega_j)p(\lambda_j|\mu_\lambda, \tau^2_\lambda))$, and $\hat{\Sigma}_{\lambda_j}$ is equal to the inverse of the second derivative of $l(\lambda_j)$ evaluated at $\hat{\lambda}_j$. If $\lambda^N_j > 0$, λ^N_j is accepted with the usual Metropolis-Hastings probability, otherwise retain λ^c_j.

5. Sample (σ^2_E, σ^2_I).

   (a) When $\varepsilon_{tj} \sim N(0, (\sigma^2_E, \sigma^2_I)z_j)$ then draw (σ^2_E, σ^2_I) from

$$p(\sigma^2_E, \sigma^2_I|\boldsymbol{Y},\boldsymbol{B},\boldsymbol{S},\boldsymbol{C}) = p(\sigma^2_E|\boldsymbol{Y},\boldsymbol{B},\boldsymbol{S},\boldsymbol{C})p(\sigma^2_I|\boldsymbol{Y},\boldsymbol{B},\boldsymbol{S},\boldsymbol{C})$$

   where

$$\sigma^2_E \sim IG\left(\frac{J_E}{2} - 1, \frac{\sum_{\{j:z_j=(1,0)'\}}(\boldsymbol{y}_j - \hat{\boldsymbol{y}}_j)'(\boldsymbol{y}_j - \hat{\boldsymbol{y}}_j)}{2}\right)\mathbb{I}\{\sigma^2_E \leq k\},$$

$$\mathbb{I}\{\sigma^2_E \leq k\} = \begin{cases} 0 & \text{if} \sigma^2_E > k \\ 1 & \text{if} \sigma^2_E \leq k, \end{cases}$$

$$\hat{\boldsymbol{y}}_j = \begin{cases} \boldsymbol{X}_{j|0}\boldsymbol{b}_{j|0} & \text{if} \quad S_j = 0 \\ \boldsymbol{X}_{j|1}\boldsymbol{b}_{j|1} & \text{if} \quad S_j = 1 \quad \text{and} \quad c_j = t^* \end{cases}$$

   and $J_E = \sum_{j=1}^{J} \mathbb{I}\{z_j = (1,0)'\}$. Similarly, draw σ^2_I with $z_j = (0,1)'$.

   (b) If $\varepsilon_{jt} \sim \sigma_j t_3$ then draw σ^2_j by

      i. Generating κ_{tj}, from a Gamma distribution $G(u_a, u_b)$ with $u_a = 2$ and

$$u_b = \frac{1}{2}\left(1 + \left(\frac{y_{tj} - \boldsymbol{X}_{tj|S_j}\boldsymbol{b}_{j|S_j}}{\sigma\sqrt{3}}\right)^2\right)$$

      where $\boldsymbol{X}_{tj|S_j}$ is a row vector denoting the tth row of $\boldsymbol{X}_{j|S_j}$ for $t = 1, \ldots, T$ and $j = 1, \ldots, J$.

ii. Generating $\sigma^2 = (\sigma_E^2, \sigma_I^2) z_j$. σ_E^2 and σ_I^2 have inverse gamma distribution with parameters (u_E, v_E) and (u_I, v_I) respectively. To draw σ_E^2, we note $u_E = J_E/2 - 1$ where $J_E = \sum_{j=1}^{J} \mathbb{I}\{z_j = (1,0)'\}$ and

$$v_E = \frac{1}{2} \sum_{\{j: z_j = (1,0)'\}} \sum_{t=1}^{T} \left(\frac{y_{tj} - \boldsymbol{X}_{tjS_j} \boldsymbol{b}_{jS_j}}{\sqrt{\kappa_{tj}/3}} \right)^2$$

σ_I^2 is drawn in a similar fashion.

6. Sample $\boldsymbol{\delta} = (\delta_0, \delta_1)$.

Draw $\boldsymbol{\delta}$ from

$$p(\boldsymbol{\delta} | \boldsymbol{Y}, C, \boldsymbol{B}, \boldsymbol{S}) = p(\boldsymbol{\delta} | \boldsymbol{S}) \propto p(\boldsymbol{S} | \boldsymbol{\delta}) p(\boldsymbol{\delta}),$$

where $p(\boldsymbol{\delta})$ is the prior distribution of $\boldsymbol{\delta}$ discussed in the main text. We use a Metropolis-Hastings method for this step . If the current value of $\boldsymbol{\delta}$ in the chain is $\boldsymbol{\delta}^c$ then a new value, $\boldsymbol{\delta}^N$, is drawn from a proposal density $q(\boldsymbol{\delta}) \sim N(\hat{\boldsymbol{\delta}}, \hat{\Sigma})$, where $\hat{\boldsymbol{\delta}}$ is the value of $\boldsymbol{\delta}$ which maximizes $\log[p(\boldsymbol{S}|\boldsymbol{\delta}) p(\boldsymbol{\delta})]$, and $\hat{\Sigma}$ is equal to the inverse of the second derivative of $\log[p(\boldsymbol{S}|\boldsymbol{\delta}) p(\boldsymbol{\delta})]$ evaluated at $\hat{\boldsymbol{\delta}}$. This new value is accepted with the usual probability.

7. Sample $(\mu_\alpha, \boldsymbol{\mu}_{\beta_1}, \boldsymbol{\mu}_{\beta 2})$.

First, draw μ_α from

$$\mu_\alpha | \boldsymbol{B}, \tau_\alpha^2 \sim N\left(\frac{\tau_\alpha^2 g_\alpha + h_\alpha \sum_{j=1}^{J} \alpha_j}{J \times h_\alpha + \tau_\alpha^2}, \frac{J \times h_\alpha + \tau_\alpha^2}{\tau_\alpha^2 h_\alpha} \right)$$

then draw $(\mu_{\beta_{1E}}, \mu_{\beta_{2E}})$ from

$$p(\mu_{\beta_{1E}}, \mu_{\beta_{2E}} | \boldsymbol{Y}, \boldsymbol{B}, \tau_{\beta_{1E}}^2, \tau_{\beta_{2E}}^2) = p(\mu_{\beta_{1E}} | \boldsymbol{B}, \tau_{\beta_{1E}}^2) \times p(\mu_{\beta_{2E}} | \boldsymbol{B}, \tau_{\beta_{2E}}^2)$$

where

$$
\begin{aligned}
\mu_{\beta_{1E}}|\boldsymbol{B},\tau^2_{\beta_1 E} &\sim N_{C_+}\left(\frac{\tau^2_{\beta_{1E}} g_{\beta_1} + h_{\beta_1}\sum_{\{j:z_j=(1,0)\}}\beta_{1j}}{J_E h_{\beta_1} + \tau^2_{\beta_{1E}}}, \frac{J_E h_{\beta_1} + \tau^2_{\beta_{1E}}}{\tau^2_{\beta_{1E}} h_{\beta_1}}\right)\\
\mu_{\beta_{2E}}|\boldsymbol{B},\tau^2_{2E} &\sim N_{C_-}\left(\frac{\tau^2_{\beta_{2E}} g_{\beta_2} + h_{\beta_2}\sum_{\{j:z_j=(1,0),S_j=1\}}\beta_{2j}}{J_{E_s} h_{\beta_2} + \tau^2_{\beta_{2E}}}, \frac{J_{E_s} h_{\beta_2} + \tau^2_{\beta_{2E}}}{\tau^2_{\beta_{2E}} h_{\beta_2}}\right),
\end{aligned}
$$

$J_E = \sum_{j=1}^{J} \mathbb{I}\{z_j = (1,0)'\}$ and $J_{E_s} = \sum_{j=1}^{J} \mathbb{I}\{z_j = (1,0)', S_j = 1\}$. Then draw $(\mu_{0I}, \mu_{\beta_{1I}} \mu_{\beta_{2I}})'$ in a similar fashion but $z_j = (0,1)$.

8. Sample $(\tau^2_\alpha, \tau^2_{\beta_{1I}}, \tau^2_{\beta_{2I}}, \tau^2_{\beta_{1E}}, \tau^2_{\beta_{2E}})'$.

   First, draw τ^2_α from $p(\tau^2_\alpha|\boldsymbol{B},\mu_\alpha)$, then draw, $(\tau^2_{\beta_{1E}}, \tau^2_{\beta_{2E}})'$ from

$$
p(\tau^2_{\beta_{1E}}, \tau^2_{\beta_{2E}}|\boldsymbol{Y},\boldsymbol{B},\mu_{\beta_{1E}},\mu_{\beta_{2E}}) = p(\tau^2_{\beta_{1E}}|\boldsymbol{B},\mu_{\beta_{1E}}) \times p(\tau^2_{\beta_{2E}}|\boldsymbol{B},\boldsymbol{\mu}_{\beta_{2E}}).
$$

   where

$$
\begin{aligned}
\tau^2_\alpha|\boldsymbol{B},\mu_\alpha &\sim IG\left(J/2-1, \frac{\sum_{j=1}^{J}(\alpha_j-\mu_\alpha)^2}{2}\right)\mathbb{I}\{\tau^2_\alpha \le a_\alpha\}\\
\tau^2_{1E}|\boldsymbol{B},\mu_{\beta_{1E}} &\sim IG\left(J_E/2-1, \frac{\sum_{\{j:z_j=(1,0)\}}(\beta_{1j}-\mu_{\beta_{1E}})^2}{2}\right)\mathbb{I}\{\tau^2_{1E} \le a_{\beta_1}\}\\
\tau^2_{2E}|\boldsymbol{B},\mu_{\beta_{2E}} &\sim IG\left(J_{E_s}/2-1, \frac{\sum_{\{j:z_j=(1,0),S_j=1\}}(\beta_{2j}-\mu_{\beta_{2E}})^2}{2}\right)\mathbb{I}\{\tau^2_{1E} \le a_{\beta_1}\}
\end{aligned}
$$

   where J_E, J_{E_s} are as defined in step 7, the function $\mathbb{I}\{\cdot\}$ is as defined in step 5 and $a_\alpha, a_{\beta_1}, a_{\beta_2}$ are calculated as described in the Priors section. Then draw $(\tau^2_{\beta_{1I}}, \tau^2_{\beta_{2I}})$ in a similar fashion but with $z_j = (0,1)$.

9. If the basis functions are exponential growth curves then μ_λ and τ^2_λ are drawn as in steps 7 and 8 above with the appropriate constraints.

TABLE 3

Estimate of posteriors means for individual probability of spiralling, $\hat{\Pr}(S_j = 1|\boldsymbol{Y})$ for all individuals classified as entity theorists (red) and as incremental theorists (blue) for three basis functions and two type of error distribution. An * or * indicates an individual classified as an entity theorist or incremental theorist respectively for whom the probability of spiralling is greater than 0.5. An estimate of the median value of the point at which the spiral begins, c_j, is given in the last column for the case when $f_j(t) = 1 - \exp\{-\lambda_j t\}$.

	$f(t) = t$		$f_j(t) = 1 - \exp\{-\lambda_j t\}$		$\hat{c}_j$
Individual	Normal	t_3	Normal	t_3	
1	0.06	0.06	0.01	0.01	0
2	0.88 *	0.44	0.99 *	0.97 *	5
3	0.27	0.28	0.22	0.09	0
4	0.03	0.06	0.01	0.01	0
5	0.16	0.25	0.20	0.09	0
6	0.01	0.03	0.00	0.00	0
7	0.05	0.08	0.01	0.01	0
8	0.10	0.11	0.02	0.03	0
9	0.01	0.04	0.01	0.00	0
10	0.20	0.37	0.22	0.07	0
11	0.06	0.04	0.00	0.00	0
12	0.12	0.13	0.02	0.03	0
13	0.11	0.24	0.15	0.04	0
14	0.76 *	0.68 *	0.77 *	0.93 *	4
15	0.22	0.22	0.05	0.14	0
16	0.55 *	0.58 *	0.54 *	0.66 *	4
17	0.05	0.12	0.02	0.02	0
18	0.98*	0.87*	0.97*	1.00*	3
19	0.97*	0.46*	0.66*	0.07	0
20	0.96*	0.86*	0.95*	0.99*	3
21	1.00*	0.92*	1.00*	0.97*	4
22	0.99*	0.97*	1.00*	1.00*	3
23	0.01	0.05	0.01	0.01	0
24	0.59*	0.66*	0.59*	0.75*	3
25	0.03	0.11	0.01	0.02	0
26	1.00*	0.98*	1.00*	1.00*	2
27	0.04	0.23	0.01	0.01	0
28	0.97 *	0.97 *	1.00 *	0.94 *	1
Average	0.63	0.68	0.64	0.61	
Average	0.17	0.19	0.14	0.14	

TABLE 4

Values of π_E and π_I used in simulation settings.

Parameter	Setting Number		
	1	2	3
π_E	0.0	0.5	0.6
π_I	0.0	0.5	0.1
$\pi_E - \pi_I$	0.0	0.0	0.5

FIGURE 7

Estimated posterior densities for the model $f_j(t) = 1 - \exp\{-\lambda_j t\}$ and $\varepsilon \sim \sigma_j t_3$. Panel (a) displays the difference in the probability of spiralling between entity theorists and incremental theorists, $\pi_E - \pi_I$. Panel (b) shows the difference in maximal performance gain between entity and incremental theorists, $\mu_{\beta_{1E}} - \mu_{\beta_{1I}}$.

(a)

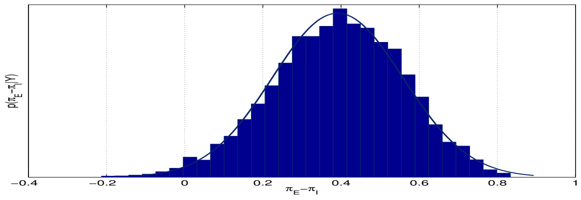

(b)

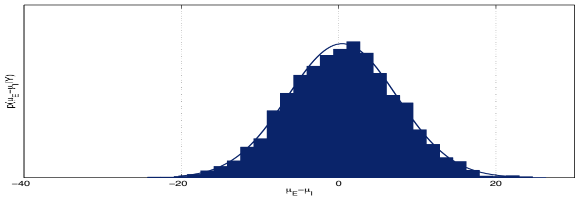

FIGURE 8

Panel (a); Posterior mean of all individual performance curves for entity theorists (red) and incremental theorists (blue) for the model with $f_j(t) = 1 - \exp\{-\lambda_j t\}$ and $\varepsilon_{jt} \sim \sigma t_3$. Panels (b) and (c) are similar plots for individuals for whom the probability of spiralling is less than 0.5 (panel (b)) and greater than 0.5 (panel (c)).

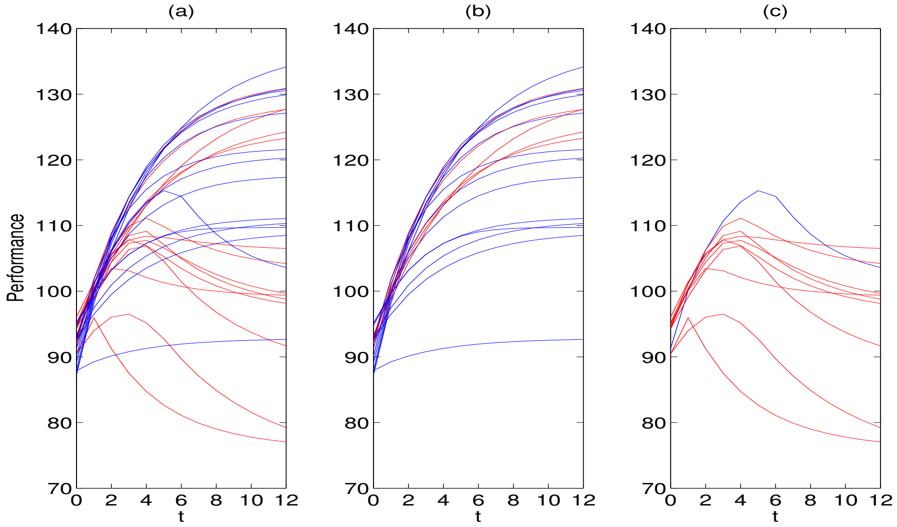

FIGURE 9

Observed performance of individual 19 and posterior mean of regression line when $\varepsilon_{jt} \sim N(0, \sigma_j^2)$, dashed (- - -), and when $\varepsilon_{tj} \sim \sigma_j t_3$, dotted (...), for $f(t)_j = 1 - \exp\{-\lambda_j t\}$.

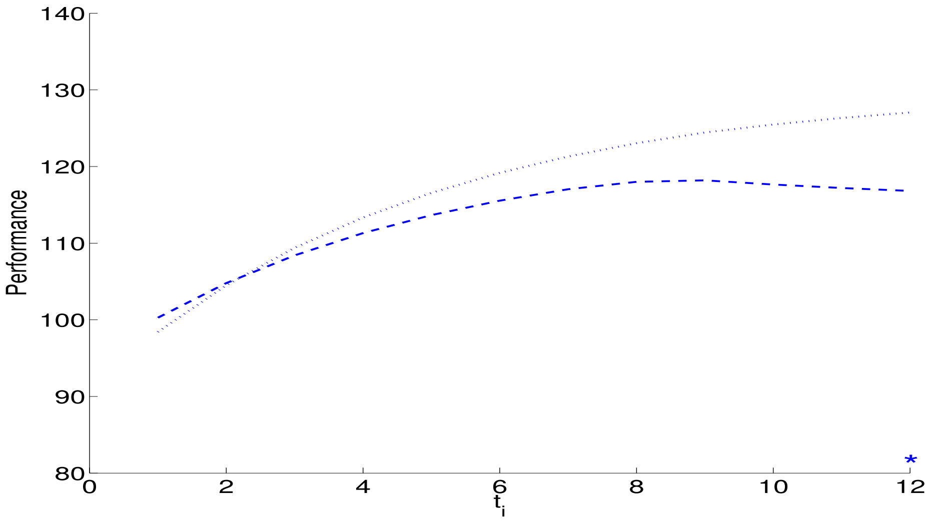

FIGURE 10

Histogram of residuals for the model given by (.4) and (.5) with $\varepsilon_{jt} \sim \sigma_j \times t_3$, and $f_j(t) = 1 - \exp\{-\lambda_j t\}$, overlaid with the density function of a t_3.

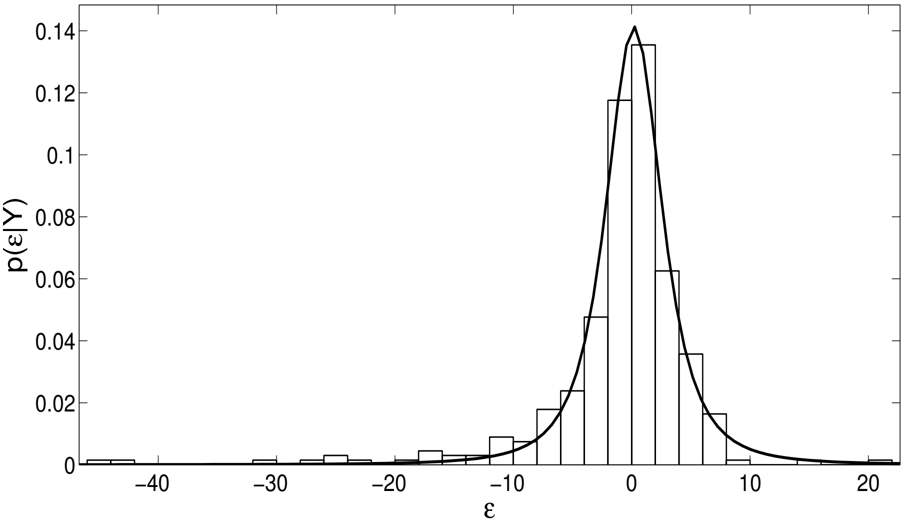

FIGURE 11

Boxplots of posterior mean estimates for 3 simulation settings with 50 realisations in each simulation. In each panel, the left boxplot corresponds to the simulation when $\pi_E = \pi_I = 0$, the middle boxplot corresponds to the simulation when $\pi_E = \pi_I = 0.5$ and the right boxplot corresponds to the simulation when $\pi_E = 0.6$ and $\pi_I = 0.1$. Panel (a) reports posterior mean estimates of $\pi_E - \pi_I$. Panel (b) reports posterior mean estimates of $\mu_{\beta_{1E}} - \mu_{\beta_{1I}}$, Panel (c) reports posterior mean estimates of σ_E/σ_I. The horizontal blue dashed line is true values.

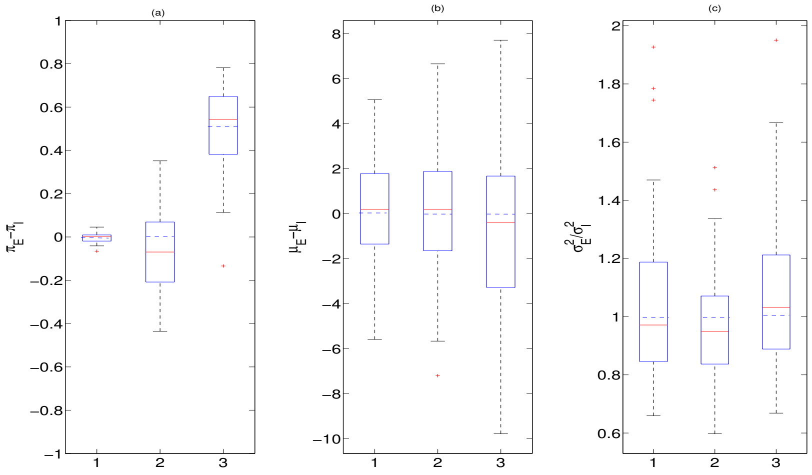

FIGURE 12

Effect of c_δ on the prior for $\boldsymbol{\pi} = (\pi_E, \pi_I)$. In panel (a), $c_\delta = 1$, in panel (b) $c_\delta = 4$, and in panel(c) $c_\delta = 10$.

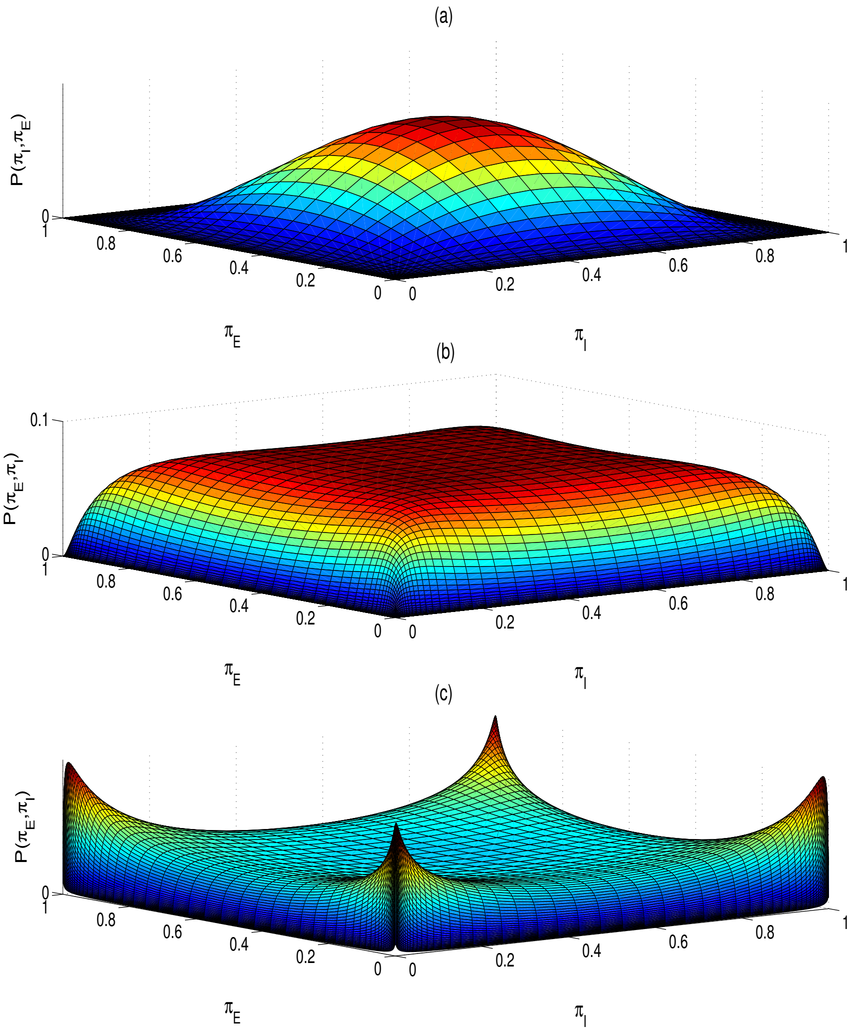

Supplement: Supplementary file 1 [file DataSheet1.pdf]
